# Secure and Sustainable Sourcing of Plant Tissues for the Exhaustive Exploration of Their Chemodiversity

**DOI:** 10.3390/molecules25245992

**Published:** 2020-12-18

**Authors:** Rhodin C. Joseph, Matheus Silva da Fonseca Diniz, Viviane Magno do Nascimento, Abraão de Jesus Barbosa Muribeca, Johan Carlos Costa Santiago, Luziane da Cunha Borges, Paulo Roberto da Costa Sá, Paulo Wender Portal Gomes, Júlio César da Silva Cardoso, Marcela Natalia Rocha de Castro, Thais Fiusa, Hervé Rogez, Sylvain Darnet, Mara Silvia Pinheiro Arruda, Milton Nascimento da Silva, Alberto Cardoso Arruda, Jean A. Boutin, Consuelo Yumiko Yoshioka e Silva, Emmanuelle Lautié

**Affiliations:** 1 Centre for Valorization of Amazonian Bioactive Compounds (CVACBA), Federal University of Pará (UFPA), Espaço Inovação, Av. Perimetral da Ciência, Belém, Pará 66095-630, Brazil; clarentz90@gmail.com (R.C.J.); matheusbiotec99@gmail.com (M.S.d.F.D.); anevivimagno@gmail.com (V.M.d.N.); herverogez@gmail.com (H.R.); sylvain@ufpa.br (S.D.); 2 Chemistry Post-Graduation Program, Institute of Exact and Natural Sciences, UFPA, Av. Bernardo Sayão, Belém, Pará 66075-110, Brazil; abraao_muribeca@hotmail.com (A.d.J.B.M.); johansantiago@hotmail.com.br (J.C.C.S.); luziane_borges22@yahoo.com (L.d.C.B.); paulorcsa@gmail.com (P.R.d.C.S.); wenderufpa@hotmail.com (P.W.P.G.); msparruda@gmail.com (M.S.P.A.); yumilton@yahoo.com.br (M.N.d.S.); 3 School of Chemistry, Institute of Exact and Natural Sciences, UFPA, Av. Bernardo Sayão, Belém, Pará 66075-110, Brazil; juliocesarjc95@gmail.com (J.C.d.S.C.); arruda.alberto@gmail.com (A.C.A.); 4 Pharmaceutical Science Post-Graduation Program, Faculty of Pharmacy, UFPA, Av. Bernardo Sayão, Belém, Pará 66075-110, Brazil; marcelarcastro@outlook.com (M.N.R.d.C.); yumikoyoshioka@yahoo.com.br (C.Y.Y.eS.); 5 Laboratórios Servier do Brasil, Estrada dos Bandeirantes, 4211, Rio De Janeiro RJ 22775-113, Brazil; thais.fiusa@servier.com; 6 Institut de Recherches Internationales Servier, 50 rue Carnot, CEDEX 92884 Suresnes, France; ja.boutin.pro@gmail.com

**Keywords:** natural products, secondary metabolites, drug discovery, analytical characterization, mass spectrometry, surface sterilization, leaf explants, plant tissue cultures

## Abstract

The main challenge of plant chemical diversity exploration is how to develop tools to study exhaustively plant tissues. Their sustainable sourcing is a limitation as bioguided strategies and dereplication need quite large amounts of plant material. We examine if alternative solutions could overcome these difficulties by obtaining a secure, sustainable, and scalable source of tissues able to biosynthesize an array of metabolites. As this approach would be as independent of the botanical origin as possible, we chose eight plant species from different families. We applied a four steps culture establishment procedure, monitoring targeted compounds through mass spectrometry-based analytical methods. We also characterized the capacities of leaf explants in culture to produce diverse secondary metabolites. In vitro cultures were successfully established for six species with leaf explants still producing a diversity of compounds after the culture establishment procedure. Furthermore, explants from leaves of axenic plantlets were also analyzed. The detection of marker compounds was confirmed after six days in culture for all tested species. Our results show that the first stage of this approach aiming at easing exploration of plant chemodiversity was completed, and leaf tissues could offer an interesting alternative providing a constant source of natural compounds.

## 1. Introduction

The diversity of the chemical compounds present in plants is immense, and sometimes this chemodiversity goes beyond what medicinal chemists can imagine and produce [1]. Within this wealth of compounds that nature produces, some possess outstanding biological activities [2]: these bioactive compounds can be of peptidic nature, like the poisons coming from many living animals or smaller molecules originating from many different chemical families and biosynthesized in plants or microorganisms, for example. They are classically named natural products (NPs), and even if they can originate from both primary and secondary metabolism, generally, the secondary “specialized” metabolism is more under focus [3]. NP are potentially active against some diseases, and studies on the origin of many of today’s drugs [4] reinforce their importance in drug discovery. In plants, secondary metabolites are present in many organs. The chemical contents of forty plants were described from their roots, flower, and leaves, with systematically a preferred organ [5,6]. It is complicated to find whether it was because of an easier collection of material (leaves, fruits, and flowers) or because the larger organs (e.g., the fruit, the bulb, or the roots) were preferred because they led to more abundant raw material. Anyway, leaves are reported as the organ of origin of many newly identified compounds (in around 30% of the plants reported in a recent survey [1]).

In drug discovery program, access to chemodiversity is crucial. In our view, the access to this existing diversity of compounds in plant tissues is incomplete when the screening concerns only plants based on ethnopharmacology data and/or oral tradition [7,8]. Indeed, these empirical data can be used performing pharmacognosy studies to explore (and predict [9]) the effects of plant secondary metabolites from which new drugs could be derived in many areas [10]. Thus, there are many reports of a classical approach attempting to discover new compounds from plant sources, including the modest contributions of the laboratories of the present authors to the field [11,12,13,14,15]. This process can be summarized as follows [16]: (i) a pharmacological target is identified, usually a receptor or an enzyme; (ii) plants are collected from the wild–with or without an ethnopharmacological rationale; (iii) they are separated into bark, leaves, fruit, flower and root, each of which can be extracted (ethanol or methanol, acetonitrile, hexane, etc.); (iv) the extracts are tested onto the target; (v) the “active” extracts use to be fractionated by reverse chromatography, and (vi) the fractions are tested again on the target. This iterative system leads to a progressive purification of the active(s) and should allow to obtain at the end of the process enough material to elucidate its structure by a combination of NMR and mass spectrometry [17]. This biologically-driven system led to interesting results, producing many papers and some patents. Unfortunately, too often, it did not result in the discovery of as many new compounds as it could have been expected or it did not result in the discovery of compounds directly usable as new drug leads. There are several reasons to explain it, but, in our view, the main ones are summarized as follows: The final compound is active but too chemically complex to be synthesized ab initio. The final compound structure cannot be resolved because there is not enough material at the end of the process because both bioguided strategies and dereplication do need quite large amount of plant material. More dramatically, there are many examples of plants leading to active extracts that are not found any more at the same location or samples where the same extract obtained from plants collected afterwards are not active anymore because of unreported existing chemotypes (see Lautié et al. for further discussion [1]). Another downside of this approach—rarely discussed—is a classic case of extracts active on the target at the first round, but then all the sub-fractions from these active fractions are inactive at the second round of testing (same target, same conditions of tests), linked to synergistic, antagonistic effects or even unexplained effects. Furthermore, when another study with a different pharmacological target comes in the picture, the whole process must start all over again (picking, extracting, testing, etc). As shown, the (re-)supply of the initial material, the plant tissue of origin of the pharmacological activity is essential [18].

Therefore, it might be interesting to try to overcome some of the difficulties described above by obtaining a secure, sustainable, and scalable source of tissues able to biosynthesize an array of those metabolites. In that sense, plant tissue culture is an established tool that has shown its interest in relation to natural products [19,20,21]. The classical approaches are usually developed after a chemical screening showed that high value target compounds accumulate in plant tissues somewhat difficult to source. In our view in vitro approaches do also make sense for the exploration of plant compounds maybe more than to produce of target compounds by optimized cell suspensions cultures. Leaves seem to be a good choice of a target tissue as it is known to synthesize a wide variety of secondary metabolites, and it is renewable for the plant itself. The culture of leaf explants is already documented [22,23,24], and it is quite an “universal” tissue, common and easily accessible for a wide majority of plant species. Being able to deal with as many plant species as possible from very diverse environments is something that is tremendously needed in order to explore natural plant chemical diversity [1]. One might think of the advantages that an approach based on the use of explants from plant parts-leaves for example-would offer. Those leaves-derived samples could be of major importance with regard to access to plant chemodiversity providing that their cells can synthesize at least some of the secondary metabolites found in extracts from plants in the wild and that these cells are still able to multiply. Indeed, they would allow constant access to this source of compounds. As a future development, one can be reminded that cells from callus-based cultures are able to multiply, can be kept frozen, are relatively fast to initiate and can be at the origin of a cell suspension if a need for scaling up is identified.

To validate such a methodology, a fair array of different plant species should be tested. We thus chose a panel of plant species from the high Amazonian biodiversity, based on two main criteria: (1) knowledge in our group of their content “in the wild” characterized by phytochemical studies carried out by UFPA researchers and (2) large botanical diversity from each other involving different botanical families.

Here we explored several ways to cultivate in vitro leaf tissues from different plant species as an alternative to iteratively (and massively?) collecting plants in the wild. We also characterized their capacities to produce secondary metabolites, taking as a reference the metabolites production of their “wild” counterpart: the leaves grown from plants harvested in natura.

One could wonder: (i) if a global procedure could be used that would allow considering any plant species and (ii) if such steps of the procedure might impair the explant’s capacity to produce secondary metabolites. Using LC-MS, it is possible to access the diversity of the compounds biosynthesized and accumulated in tissues of the biological samples. Thus, the overall capacity of the explants to produce secondary metabolites has been characterized at every step of the process, while monitoring selected compounds characteristic of a given plant synthetic pathways. We have shown that most of the samples produce a large diversity of compounds, based on mass spectrometry ion surveys, that is surprisingly similar to the one produced by leaves from the wild. Obtaining such wide diversity of compounds in each sample in culture with this approach could open new avenues to access plant chemical diversity.

## 2. Results and Discussions

### 2.1. In Vitro Culture Establishment

One important step for aseptic culture establishment is the surface sterilization of the explants. First, several ways to produce surface-sterilized leave explants were explored. We wanted to test if it was possible to design a “universal” methodology, “universal” in the sense that the methodology could be used to establish in vitro cultures from (almost) any plant species. This hypothesis was quite challenging if we refer to what is usually done. Indeed, classically, the conditions of the surface sterilization procedure are optimized in function of the plant species [25,26,27] and the susceptibility of its tissues in order to obtain explants with both the best decontamination rate and with tissues having a good viability.

**Figure 1:**
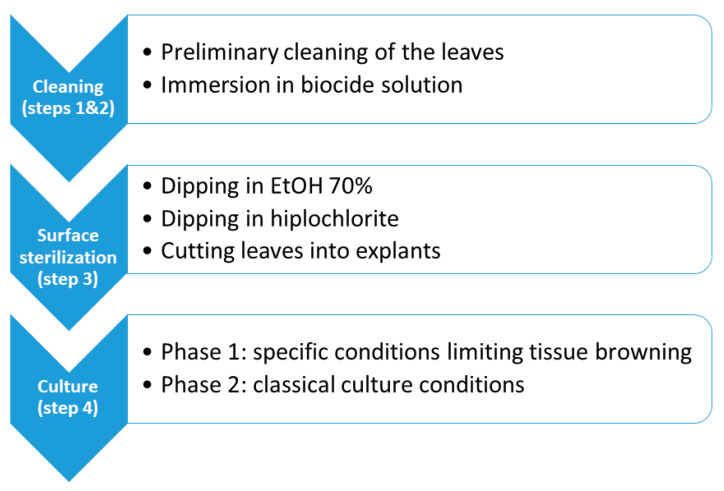
Workflow of a standardized procedure, including surface sterilization from leave samples collected in natura.

As a consequence, a standardized method presented in Figure 1 was developed and tested. Then, considering that we needed to attend very diverse plant species, several levels of flexibility of the methodology were introduced in order to increase the probabilities for this methodology to be “universal”. First, during the surface sterilization procedure: its conditions were “standardized” for all the tested species, but at the same time, these conditions were flexible in terms of the time of exposure, depending on if the plants belong to herbaceous or tree species (same steps but different times of application). Then, the second level of flexibility was introduced for some specific plant species for which the basic procedure was slightly adapted. The third level was to develop an alternative method to obtain surface-sterilized leave explants through the culture of axenic plantlets, as an alternative source of sterile plant tissues, in the case the first sterilisation process failed.

### 2.2. Efficiency and Impact of the Surface Sterilization Procedure on Leave Explants

**Figure 2:**
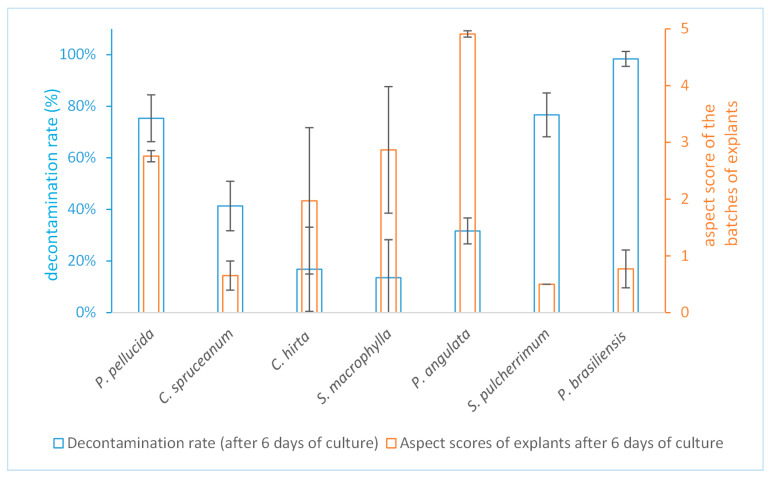
Evaluation of the efficiency and impact of the surface sterilization procedure on the leaf’s explants (for each species, the procedure was realized on batches of 120 explants and repeated three times).

Regarding the efficiency of the surface sterilization procedure, the decontamination rate obtained for the tested species is illustrated in Figure 2.

The level of efficiency of the procedure seems to depend on the species with decontamination rates ranging from 97% (for *Phyllanthus brasiliensis)* to 14% (for *Swietenia macrophylla)*. For some species, the results are variable in function of the repetitions as they can be influenced by the season of the harvest as well as the “maturity” of the leaves collected, especially for perennial species with leaves that deal with microorganisms for longer periods [28,29]. As expected, it was not easy to meet the challenge for the chosen species because of their diversity. As mentioned, they were diverse in terms of initial contaminants loads or diverse in terms of leaf anatomy, e.g., abundant trichomes on leaves of *Clidemia hirta* limiting the wetting process and, consequently, the surface sterilization process. In any case, we managed to surface-sterilized leave explants with a decontamination rate that allows working (>15%) for five plant species upon the eight collected in natura and only one species (*Vatairea guianensis*) did not show any decontamination in the condition set in this methodology.

Since disinfectants can be toxic to plant tissues, so it seemed important to evaluate the impact of the procedure. The viability of the explant’s tissues should be preserved as much as possible for the sake of the future culture if one wants to induce callus, for example. Here, this parameter was evaluated through a rational scale of scores based on the visual aspect of the leave explant tissues. Instead of qualitative scoring systems (i.e., more or less fresh and green ≠ withered and brown), the quantitative scale that we used allowed the evaluation of each contamination-free explants individually. Then, an average of the individual scores of each explant could be calculated, and this average would characterize the whole batch of explants. It also allowed us to compare the batches between one another based on this average. This is a better way to evaluate a procedure, taking into account the individual variability between explants linked to the variability between leaves and/or source plants. It also permitted us to limit subjectivity during the evaluation of the aspects of the explants and to standardize the evaluation between the different operators and from one repetition to the other.

**Figure 3:**
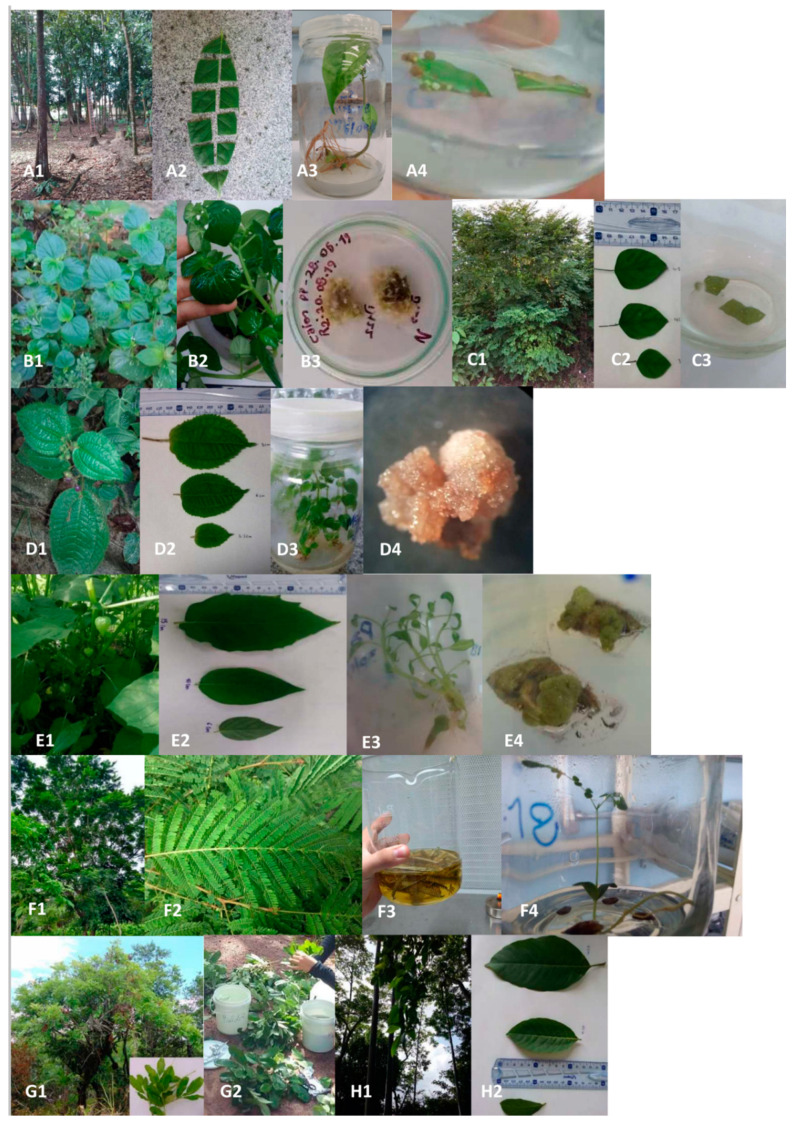
Establishment of in vitro leaf tissue culture for eight plant species: from leave samples collected *in natura* to in vitro cultures. *Swietenia macrophylla*’s trees (**A1**), leaf and explants (**A2**), axenic plantlets (**A3**) and calli (**A4**); *Peperomia pellucida*’s plants and leaves (**B1**,**B2**), callus (**B3**); *Phyllantus brasiliensis*’s plant (**C1**), leaves (**C2**) and explants on solid medium (**C3**); *Clidemia hirta*’s plant (**D1**), leaves (**D2**), axenic plantlets (**D3**) and callus (**D4**); *Physalis angulata*´s plant (**E1**), leaves (**E2**), axenic plantlets (**E3**) and calli (**E4**); *Stryphnodendron pulcherrimum* ´s tree (**F1**), leaves (**F2**), surface sterilization process (**F3**), axenic plantlets (**F4**); *Vatairea guianensis*´s tree and leaves (**G1**) precleaning step of the leaves (**G2**); *Calycophyllum spruceanum*´s tree (**H1**) and leaves (**H2**).

It was also interesting to compare the impact of this “standardized” procedure on the different species. As illustrated in Figure 2, the impact was quite different depending on the species. The average scoring ranged from very good for *Physalis angulata* explants (4.9 ± 0.08) to very poor for *Stryphnodendron pulcherrimum* explants (0.5 ± 0.001). This last result can be related to the small size of the leaf explants of *Stryphnodendron pulcherrimum* due to the initial size of its leaflets (~ 2 mm width as shown in Figure 3-F2). In fact, the impact is higher on small explants because of the higher proportion of wounded tissue. It also makes it more difficult for the explant tissue to recover after the procedure.

Based on an acceptable impact of “less than two-thirds of the explant area visibly affected” (corresponding to aspect scores > 2 in our scale), four plant species resulted having leaf explants that responded to the standardized decontamination protocol in a way that allows developing cultures afterward (as illustrated in Figure 3).

We manage to establish the culture of a fifth species, *Phyllanthus brasiliensis*. The efficiency of the surface sterilization and impact of the procedure are often related (and for this reason, they are represented together in Figure 2). Indeed, conditions of the surface sterilization procedure leading to high efficiency in terms of decontamination may have a strong impact on the tissues, as it is the case for *Phyllanthus brasiliensis*. In this sense, as the decontamination rate for this species was very high, we could slightly adapt the procedure to “milder” surface sterilization conditions (5 min instead of 20 min in hypochlorite) and could rise the aspect score to 2.5 with still a decontamination rate of 23%. This optimization of the conditions from the standard procedure illustrates the second level of flexibility of the methodology presented in Section 3.1.

Another interesting point is related to oxidative browning, which could consist of a severe limitation in tissue culture [30,31] Woody species are supposed to be more prone to developing it than herbaceous species as we experienced, for example, with *Swietenia macrophylla* and *Calycophyllum spruceanum* leaf explants. In fact, we designed the first phase of the culture step (in Figure 1) to reduce as much as possible the oxidative browning of the wounded tissues. Upon cultures, conditions that are known to contribute reducing the “browning” of tissues are favoring antioxidant conditions as well as conditions contributing to lower activity of enzymes like polyphenoloxidase, slower metabolism, and reduced available O_2_ [32,33,34,35]. Therefore, during the three first days of the culture, conditions such as ascorbic acid and activated charcoal in the media medium in liquid form as well as the culture at lower temperature and under darkness were set up. With these conditions, oxidative browning was controlled successfully for *Swietenia macrophylla* but not for *Calycophyllum spruceanum* leaf explants.

### 2.3. Alternative Method to Produce Sterile Leave Explants

The culture of axenic plantlets can be initiated from fruits and seeds harvested *in natura* and surface sterilized. This could be an interesting alternative, especially for plant species that responded poorly to surface sterilization of leaves. Furthermore, once initiated, these cultures can be maintained by micropropagation, which allows obtaining many shoots with leaves originating from an initial reduced number of seeds. In our case, in relation with the availability of fruits and seeds material, axenic plantlets from four species were obtained (Figure 3) from both tree and shrub species (*Swietenia macrophylla, Stryphnodendron pulcherrimum*, *Clidemia hirta* and *Physalis angulata*).

Promising results were obtained for example with *Swietenia macrophylla* with fast germination of seeds: the first ones germinated in 5–7 days, while the average germination rate was 83% (± 6%) and the average decontamination rate 70%. The first leaves appear after 21 days of culture, while an average of 10 explants per plantlets could be obtained after 45 days. This alternative can also generate explants with a better aspect of leaf explant tissues linked with their viability confirmed by average aspect score of 4.0 (± 0.77) because they did not go through a procedure of surface sterilization. Improved results in terms of the aspect of the leaf explants were also obtained with *Clidemia hirta* (average aspect score of 3.3 ± 0.51). For this species, the leaf explants were excised from axenic plantlets obtained after 2 months. The fruits of *Clidemia hirta* present many seeds in general but if one considers both germination rate and loss with contamination, on average, three axenic plantlets could be actually grown per fruits with their surface previously sterilized (average germination rate of 20% ± 5%, and decontamination rate of 73% ± 18%).

**Figure 4:**
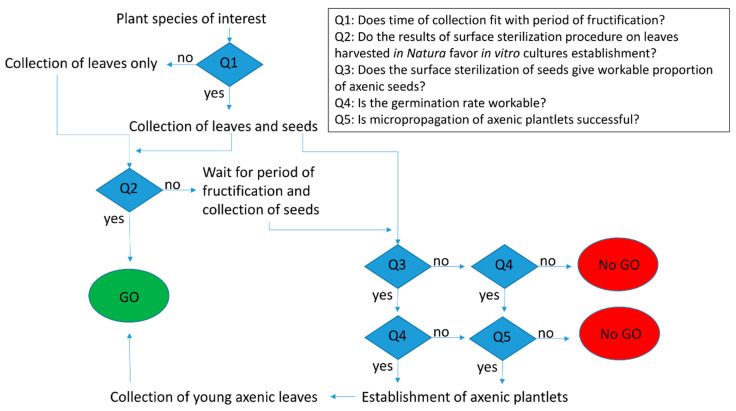
General flowchart for the establishment of in vitro leaf tissue culture (GO: cultures established; No GO: cultures not established).

Of course, this alternative method is more time consuming and involves more resources than harvesting leaves in natura, but it also allows us to generate pools of plantlets that will be an endless source of aseptic explants. As a consequence, it should be considered in combination with the previous strategies (applying the procedure of leaves surface sterilization with possible adaptations) for the plant species for which these strategies were not so propitious or favorable (ex: low decontamination rate and/or poor tissues aspect). In summary, we propose in Figure 4 a flowchart for the establishment of in vitro leaf tissue culture as “universal” as possible. We tried to generalize, from our results, the whole process considering the different questions that will drive the choices among the several alternatives presented here: (i) if the procedure of surface sterilization on leaves harvested in natura gives favorable results, (ii) if the decontamination rate of seeds and their germination rate are workable, (iii) if it is feasible to micro-propagate the plantlets generated. Furthermore, if the species of interest consist of tree species, it may be advisable to wait for the period of collection to coincide with fructification, as it can be less easy for some woody species to get favorable conditions for the establishment of in vitro cultures [36].

A few reviews mention general protocols for the establishment of in vitro cultures, most of them dealing with the adaptation of these procedures in the up-scaling context [19,37]. Nevertheless, it is classical to think that universal surface sterilization procedures that could be applied to all plant species would be impossible to develop [38]. Indeed, we experienced some difficulties when it came to establish standardized conditions for in vitro cultures from in natura biological material instead of greenhouse-grown material with the controlled sanitary regime. But our objective was to ease the access to plant tissues with a great chemodiversity, i.e., ideally collected from any environment. Some of the plant species clearly responded differently from one another with regard to in vitro culture. Anyway, our results show that a combination of alternatives could overcome the limitations due to the characteristics of specific plant species. As illustrated in Figure 3, with the plant material that we manage to collect, in vitro cultures were established successfully for six species. Back to the scheme of Figure 4, we can infer that cultures could not be established only in cases when the surface sterilization procedure after harvesting leaves in natura gives unfavorable results AND the seeds cannot easily be decontaminated, AND they did not germinate well OR if the micropropagation is inefficient. Of course, this design should be applied to a larger pool of species for validation. However, applying the methodology designed in Figure 4, with full access to plant material like fruit and seeds, we consider that it is feasible to establish in vitro cultures for about 2/3 of the intent plant species, even if the environment is highly contaminated as during the Amazonian rainy season or considering that some species could have recalcitrant seeds.

The second part of our study tried to bring a light on the chemodiversity of explants tissues in cultures through (i) the evaluation of some possible changes in the metabolite composition of leaf tissues during the establishment of in vitro culture through the presence/absence of some selected metabolites and (ii) the evaluation of the overall diversity of compounds in leaf explants in cultures through their chemical profiles.

### 2.4. Evaluation of the Impact of the Establishment of in Vitro Cultures on Selected Leaf Metabolites

Our goal was to allow easy and constant access to plant chemical diversity. For this reason, in the first stage of our approach, we needed to establish in vitro leaf tissue cultures that would allow avoiding multiple collecting campaigns in the wild. To progress along this line, it was necessary to assess the presence of metabolites at each step of the procedure, including cleaning, sterilization, and culture. To do so, we developed analytical tests based on the use of mass spectrometry. For each species, a dozen reference metabolites produced by different synthetic pathways of plants were selected. We chose this panel of compounds among the ones we were familiar with, as shown in Table 1. Here, our hypothesis to test was that those compounds—or most of them—should be produced in detectable quantities, up to the callus stage.

**Figure 5:**
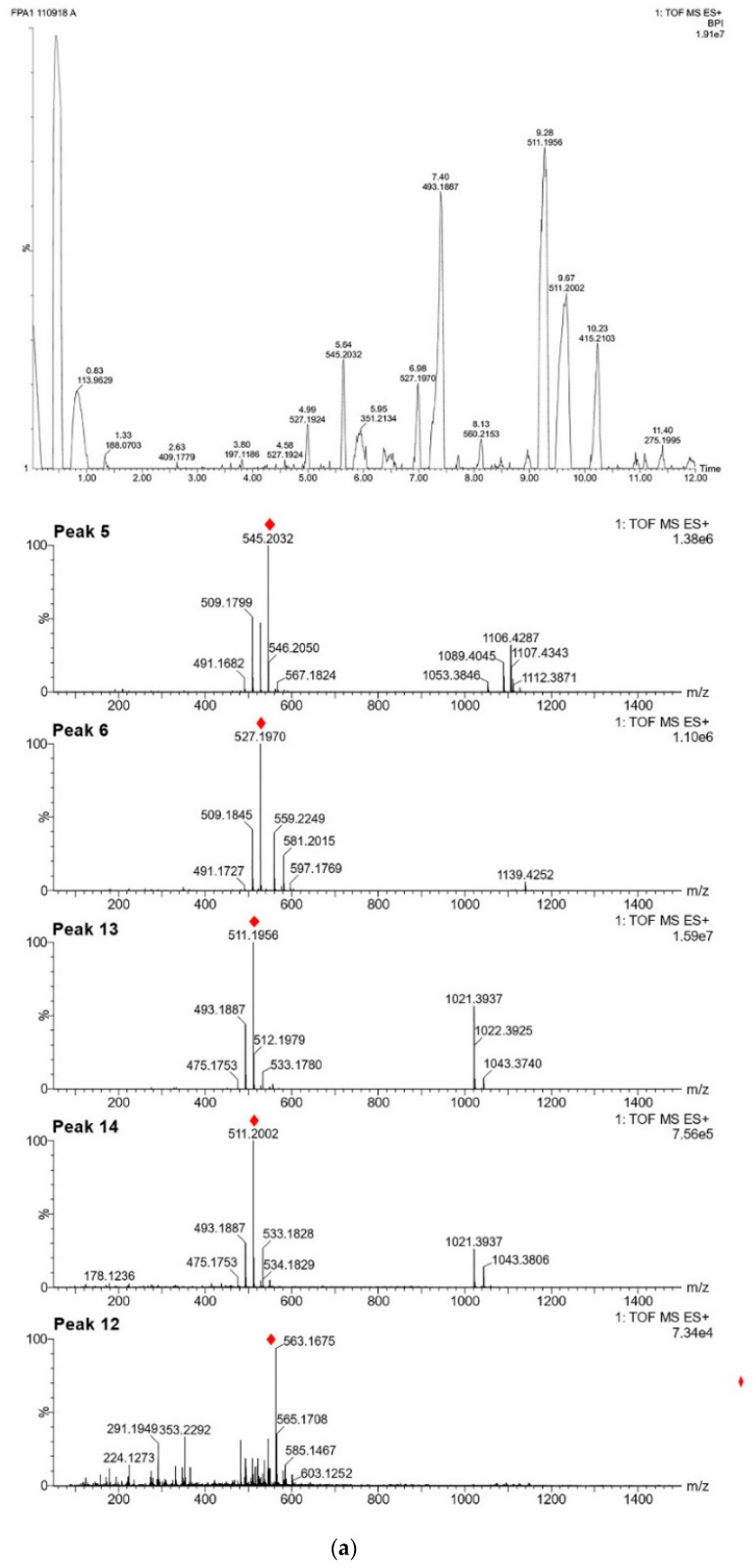

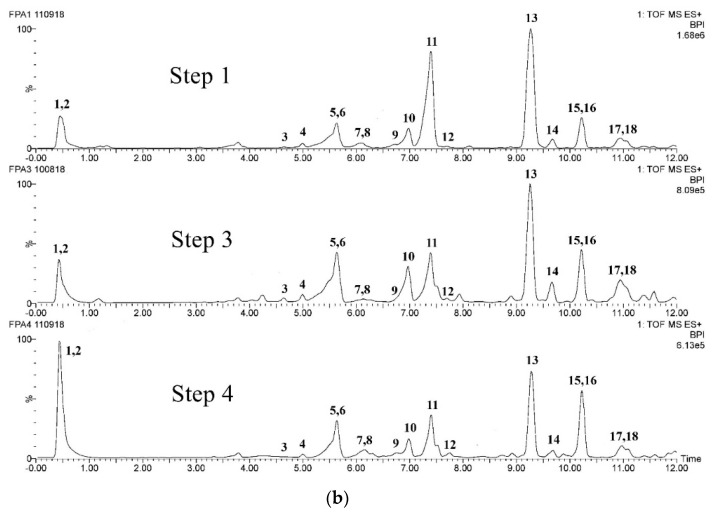
Base peak ion chromatograms of the leaf explants from *Physalis angulata* (**a**) with mass spectra extracted for several peaks of interest (red symbol indicates the molecular ion) (**b**) comparing steps 1 to 4 (the numbers in bold correspond to the compounds detailed in Table S1).

**Figure 6:**
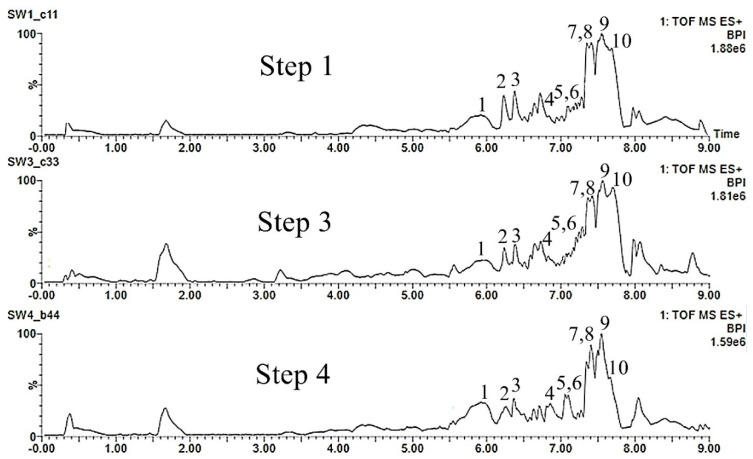
Base peak ion chromatograms comparing steps 1 to 4 of the leaf explants from *Swietenia macrophylla.*

**Figure 7:**
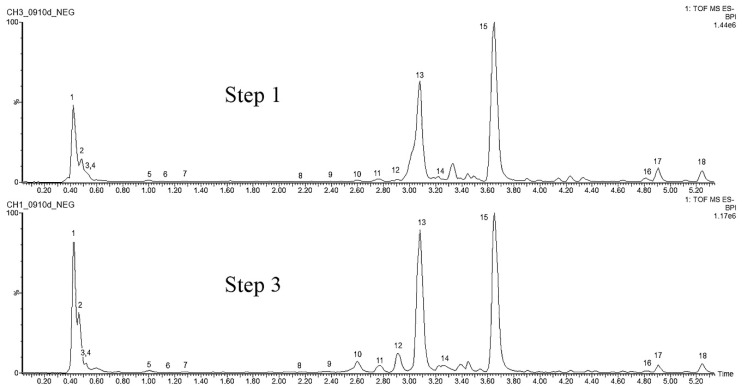
Base peak ion chromatograms comparing steps 1 to 4 of the leaf explants from *Clidemia hirta.*

**Figure 8:**
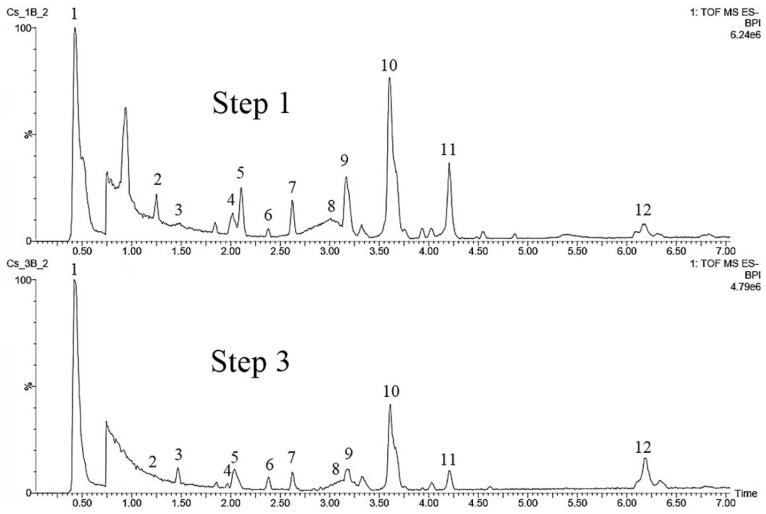
Base peak ion chromatograms comparing steps 1 to 4 of the leaf explants from *Calycophyllum spruceanum.*

**Figure 9:**
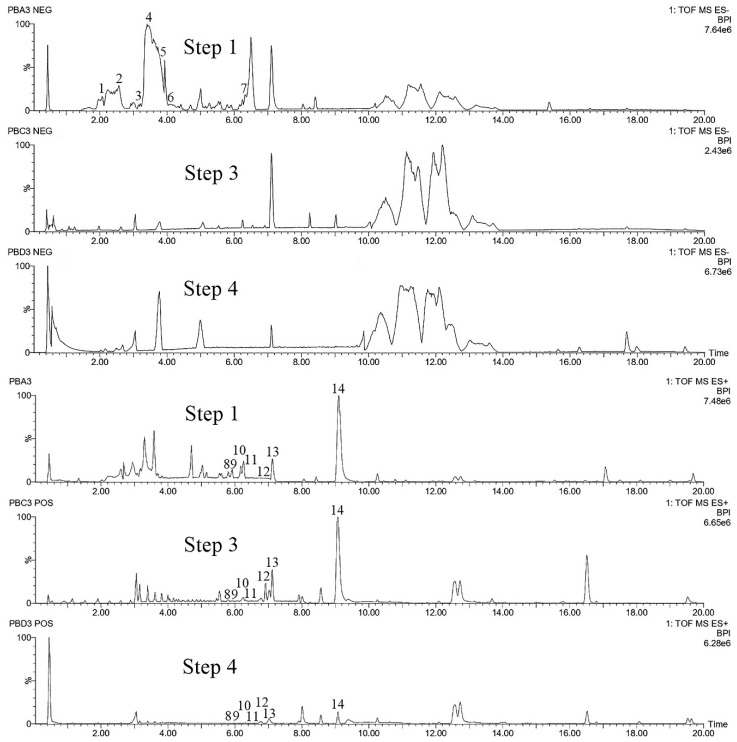
Base peak ion chromatograms comparing steps 1 to 4 of the leaf explants from *Phyllanthus brasiliensis.*

**Figure 10:**
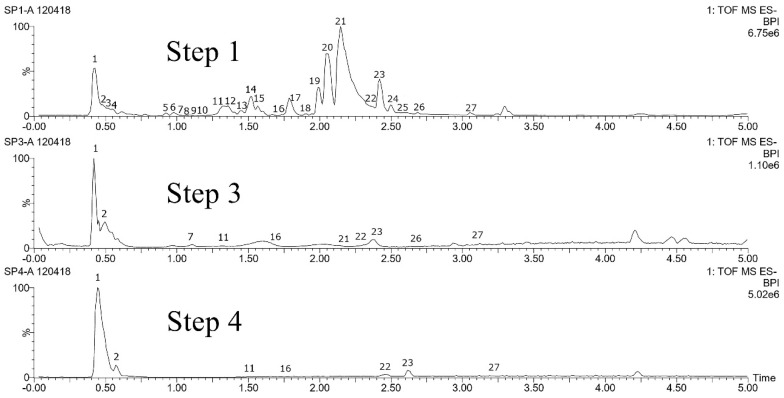
Base peak ion chromatograms comparing steps 1 to 4 of the leaf explants from *Stryphnodendron pulcherrimum.*

For each species, we performed analyses of leaf samples, which have grown in natura (step 0), in order to obtain a profile of secondary metabolites biosynthesized in natural conditions before any treatment. The identification of compounds both at step 0 or after consecutive in vitro culture establishment procedure (steps 1–4) were carried out using their retention times and mass spectra provided by TOF-MS. These data were compared with those of authentic chemical standards when available (*Pyllanthus brasiliensis* and *Swietenia macrophylla*) or with a databank of substances present in the species, genus, or family (present on HMDB, MoNa, and METLIN database, for example). Some unknown compounds were considered as other markers in this work because they were present in step 0 in several replicates. Figure 5, Figure 6, Figure 7, Figure 8, Figure 9 and Figure 10 and Figures S1 and S2 in Supplementary Material show base peak ion chromatograms (BPI) of leaf explant extracts from each plant species, collected after each step of the procedure. Nevertheless step 2 will be presented in the figure only when it differs from step 1 and 3. For more details about the characterization of chemical constituents on the plants studied here, see Supplementary Material: Tables S1–S8. Mass spectra extracted for several interesting peaks of each species chromatograms are presented in Figure 5a and Figures S4–S11.

#### 2.4.1. *Physalis Angulata* L.

For the analysis of the samples of *P. angulata*, peaks were monitored in the positive mode: Figure 5a shows the base peak ion chromatogram and several mass spectra extracted for some peaks of interest. A total of eighteen substances, listed in Table S1, were used as target metabolites to evaluate the impact of the culture procedure steps on secondary metabolic production. The physalins are considered biomarkers of species from the genus *Physalis*. Thus, among the eighteen compounds, eight physalins were considered as targets, four of them compared with substances previously isolated by our group: physalins D (**5**), G (**6**), B (**13**) and isophysalin B (**14**) [39] and four ions were detected and attributed for compounds **4**, **10** and **11** (isomers of physalin G) and **12** (physalin H) [49]. The BPI analysis shows all peaks considered for process evaluation at steps 1 to 4 (Figure 5b), which allows us to infer that the procedure has not induced visible changes in the composition of these metabolites.

#### 2.4.2. *Swietenia macrophylla* King

Ten secondary metabolites were used in the comparison of the BPI of *S. macrophylla* leaf explants extracts. These marker compounds belong to the class of limonoids, which are taxonomic markers of the species. The mass spectra of these compounds were compared with those obtained from the substances isolated and identified by our group [12,40,41]. The chromatographic profiles of the samples (Figure 6) associated with the four steps were compared to obtain a preliminary evaluation of the procedure. The ions corresponding to all ten limonoids were identified in all samples (steps 1 to 4), as listed in Table S2. The culture establishment procedure did not seem to influence the metabolic production of these compounds.

#### 2.4.3. Clidemia hirta (L.) D. Don

Eighteen target metabolites, listed in Table S3, were selected for the analysis of *C. hirta* samples to evaluate the impact of the culture establishment procedure. Among them, ten were identified by comparison with the data described in the literature. The compounds referring to peaks **1**, **2**, **3**, **13,** and **15** when compared to the literature correspond to quinic acid, gallic acid, ellagic acid, rutin, and quercetin, respectively. Peaks **4**, **5**, **6**, **7** and **9**, presented characteristic *m/z* ratio of the ellagitannins (hydrolyzable tannins), according to Engström [50]. Abdellaoui et al. [51] also identified the compounds referring to peaks **2** and **3** (ellagitannins precursors), and **4**, **5**, and **7** in the ethyl acetate extract of *C. hirta* roots corroborating the data found here.

All the eighteen markers were detected in steps 1 and 3, suggesting that the procedure itself did not induce alteration of these kinds of metabolites. In step 4 of the culture establishment procedure, the profile is very similar, as it can be seen in Figure 7, except for compound 8 that was not detected.

#### 2.4.4. *Calycophyllum spruceanum* (Benth.) Hook.f. ex K. Schum.

The peaks for the twelve substances, listed in Table S4 and shown in Figure 8, were monitored in the negative mode to evaluate the impact of the steps of the culture establishment procedure on *C. spruceanum* samples. Six peaks were identified based on comparison with literature [52,53,54,55,56] data as: **1**-quinic acid; **2**-caffeoylquinic acid; **3**-(epi)catechin dimer; **8**-(epi)catechin trimer monoglycoside; **9**-quercetin 3-*O*-α-arabinopyranosyl (1→6) *p*-glucopyranoside; **10**-kaempferol 3-*O*-β-D-glucopyranosyl (1→2)-β-D-xylopyranoside. By comparison of BPIs of steps 1 to 4, it can be observed, based on the target compounds and the global BPI profiles, that the cleaning and the surface sterilization procedures do not seem to alter the samples composition profile for this plant species.

#### 2.4.5. *Vatairea guianensis* Aubl.

For *V. guianensis* samples, fifteen target metabolites, listed in Table S5**,** were selected to evaluate the culture establishment procedure in positive mode. Eleven compounds could be identified by comparison with the data described in the literature. Compounds referring to peaks **4**, **7**, **11**, **12**, **13**, **14** and **15** have already been identified in the same species [43,44], while compounds **1**, **2**, **3** and **10** were identified based on the exact masses compared to those described in the literature [57,58,59,60] for compounds already reported in the Fabaceae family. Compounds **5**, **6**, **8,** and **9** could not been identified. Base peak ion chromatograms for the culture establishment procedure (steps 1 and 4) are shown in Figure S1. Chromatogram analysis showed that all markers described were detected in steps 1 and 3, suggesting that the procedure did not induce visible alterations.

#### 2.4.6. *Peperomia pellucida* (L.) Kunth

For the analysis of the samples of *P. pellucida*, nine compounds could be characterized while detected in positive mode, which diversity of chemical groups is showed in Table 2. Exemplification of the way the characterization was realized is given for several compounds in Schemes S1–S6 of the supplementary material. Further information could be obtained upon request. These compounds described in Table S6 were used as target metabolites to evaluate the impact of the culture establishment procedure. Of these, five matched with known compounds from the genus or species when compared with data described in the literature [46,61,62] or isolated standard (pellucidin B). Base peak ion chromatograms for the different steps of the procedure steps 1 to 4 are shown in Figure S2. Based on the chromatograms, all marker compounds were detected in steps 1 and 3, suggesting that these procedures (cleaning and surface sterilization) did not induce immediate alterations. On the other hand, after 6 days-culture (step 4) some metabolic changes can occur as several marker compounds were not observed any more: ions at *m/z* 565.1553, 197.1172, 197.0818 and 389.1960 assigned to the compounds isoschaftoside (**1**), a non-identified compound **2**, 2-hydroxy-4,6-dimethoxyacetophenone (**3**) and pellucidin A (**7**), respectively. Although no qualitative differences were observed between steps 1 and 3, a variation in the relative intensity of ions was observed for compounds **6** (pellucidin B) and **7** (pellucidin A), as shown in Figure S2, starting at step 1 with a ratio of approximately 1:1 and with a slight decay at step 2 and higher at step 3, as if there was a progressive decline on compound **6** concentration. A possible explanation for this would be the occurrence of a 1,5-sigmatropic rearrangement in the cyclohexene ring of pellucidin B (*m/z* 387.1808), favored by acid culture medium, which could explain the formation of pellucidin A (*m/z* 389.1960).

#### 2.4.7. *Phyllanthus brasiliensis* (Aubl.) Poir.

Fourteen compounds, listed in Table S7, were selected in negative and positive ionization modes (LC-MS) and used as target compounds to evaluate possible changes in the metabolism of *P. brasiliensis* during the in vitro culture establishment procedure. Compounds **1** to **7** ionized better at negative mode and were characterized as flavonoids [63,64,65,66] except for compound 7 that is a lignan (arabelline) according to data comparison, as described previously, or as unknown flavonoids (**4** and **6**). The total ion chromatograms of the different steps of the culture establishment procedure, shown in Figure 9, were compared, and the results showed that none of the selected marker compounds observed in steps 1 and 2 at the negative mode are detected in steps 3 and 4. In this species, the procedure can induce some kind of modifications in the metabolic composition of the leaf tissues. Nevertheless, the BPIs profiles after the 4th min of the run are globally similar qualitatively.

In positive mode, some other marker compounds (**8** to **14)** ionized better. They belong to lignan class, except for **11** that is a flavonoid (kaempferol). The lignans were previously isolated and identified by our group from the methanolic extract of *P. brasiliensis* leaves and considered as reference standards at this work. Regarding the BPIs profiles (Figure 9), differences between steps 1 and 3 are still perceptible at the positive mode of ionization, especially for compounds from the beginning of the BPI. But for the lignans identified as markers, it was observed that they were all detected after each step, unlike the flavonoids. After step 4, the composition of the sample differs quantitatively mainly regarding compound **14** (justicidin B), which presents low intensity of ions when compared to the other steps (1 to 3).

#### 2.4.8. *Stryphnodendron pulcherrimum* (Willd.) Hochr

To evaluate the culture establishment procedure on samples of *S*. *pulcherrimum* twenty-seven compounds, listed in Table S8, were used as markers. Of this total, fourteen were identified as tannins through data compared with those described in the literature [63,67] or with spectra libraries (GNPS, METLIN, and HMDB). After comparing the samples in natura with the samples collected at each step of the in vitro culture establishment procedure of *S. pulcherrimum* on the LC-MS directed metabolic study, significant changes were observed. Based on total ion chromatograms shown in Figure 10, the 27 marker compounds were detected in step 1. A progressive decay of the number of markers was observed in the samples collected after steps 2, 3, and 4, respectively 52%, 37%, and 26%. Modifications in the profile begin as early as step 2, with the soaking of the leaves in the biocide solution, but a more drastic change in the overall profile is observed at step 3 after the surface sterilization step. As noticed previously (Section 3.2), the very small size of the leaflets of *S. pulcherrimum* and of its explants results in a very high proportion of wounded tissue in which cellular content seems affected. The tissue proves to be unable to recover even after several days in conditions more favorable as proven by step 4′s BPI (Figure 10). Furthermore, in step 4 only 26% of the initial marker compounds are detected in relation to the reference samples, and none of these are tannins. Tannins are produced by the secondary metabolism of plants against attack by insects and microorganisms, playing an important role in *Stryphnodendron* species, with about 40% of secondary metabolites in leaf composition [68,69]. Studies report that tannins have the property to interact with substances [70] such as PVP by complexation mechanism, and this reagent is part of the composition of the culture media used in the procedure.

#### 2.4.9. Conclusions of the Leaves Explants Analyses on All Species

The eight plant species selected in this research belong to seven distinct botanical families, as shown in Table 1. They have in common their main occurrence in tropical and subtropical regions. These species have economic importance and/or use in traditional medicine. The main classes of substances identified in the leaf samples collected in natura and used as marker compounds are listed in Table 2. They belong to a wide variety of metabolites like triterpene derivatives such as limonoids and physalins, cinnamic acid derivatives such as flavonoids, isoflavonoids, lignans, ArC2 dimers, phenolic acids, and condensed tannins, as well as gallic acid derivatives such as ellagitannins. Indeed, it is possible that the choice of the compounds acting as “markers” affects the results of the evaluation of the procedure. In *Phyllanthus brasiliensis* tissues, for example, the presence of compounds at steps 3 and 4 seems to depend on the kind of compounds. Nevertheless, the choice has been made previously, and the number of selected markers is representative of the overall number of peaks that can be observed in BPIs. After the pre-cleaning (step 1), there were no changes in the composition regarding the markers identified in the leaves in natura for all species studied. After cleaning and surface sterilization of the leaves (steps 1–3), six of the eight species maintained the composition of these markers, and only *Phyllanthus brasiliensis* and *Stryphnodendron pulcherrimum* species showed leaves explants with their composition affected in several of the marker compounds. In these two same species, not only the composition in these metabolites but also their biosynthesis seemed affected: several days of culture of the leaf explants did not restore the initial composition and after step 4 (in vitro leaf tissue culture) about 50% and 25% only of the marker compounds of *Phyllanthus brasiliensis* and *Stryphnodendron pulcherrimum* were detected. Interestingly the results of the analyses based on the markers detected at step 4 support the evaluation realized upon the scoring of the aspects of the explants after 6 days of culture, especially in the case of *Phyllanthus brasiliensis* and *Stryphnodendron pulcherrimum* (Figure 3). Indeed, when the tissues of the explant are no longer viable, only few compounds are detected in their extracts. From a methodological point of view, it also confirms that this simple and affordable standardized scoring system based on aspect is reliable as a preliminary screening. Finally, based on the evaluation of the chemical profiles of explants collected at step 4, four species out of six still show a diversity of compounds in leaf explants in cultures.

#### 2.4.10. Analyses of Metabolites from Explants Originating from Axenic Plantlets

The purpose of the analyses was to evaluate if the alternative procedure of culture establishment induces changes in the diversity of compounds in leave explants and how. This time leaves from axenic plantlets originating from fruits and seeds of tree and shrub species were excised (step 1′) and explants analyzed after growing them for 6 days in vitro (step 4′).

**Figure 11:**
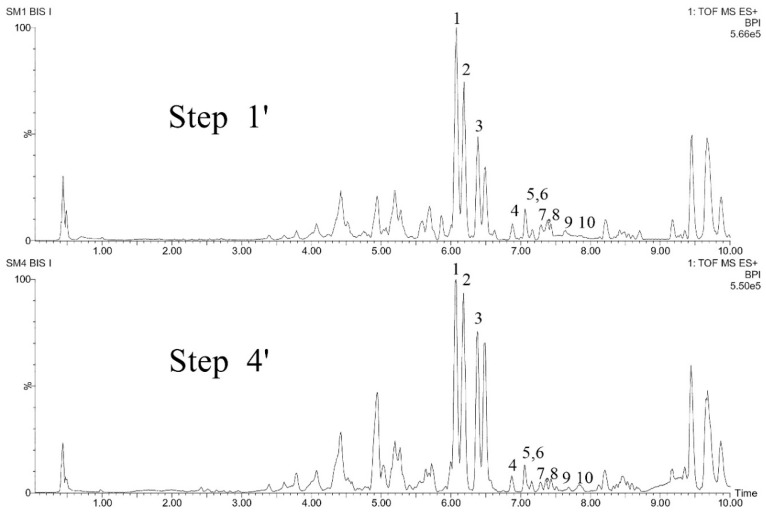
Base peak ion chromatograms comparing steps 1′ and 4′ of the seedling leaf explants from *Swietenia macrophylla.*

Figure 11 shows total ion chromatograms for step 1′ and step 4′ for *Swietenia macrophylla* explants. As it can be observed in this figure, the same ten marker compounds identified in leaves collected in natura were detected in leaves from axenic plantlets germinated in vitro. They were still detected in explants cultivated for 6 days. Furthermore, their overall profiles are globally very similar for both steps.

**Figure 12:**
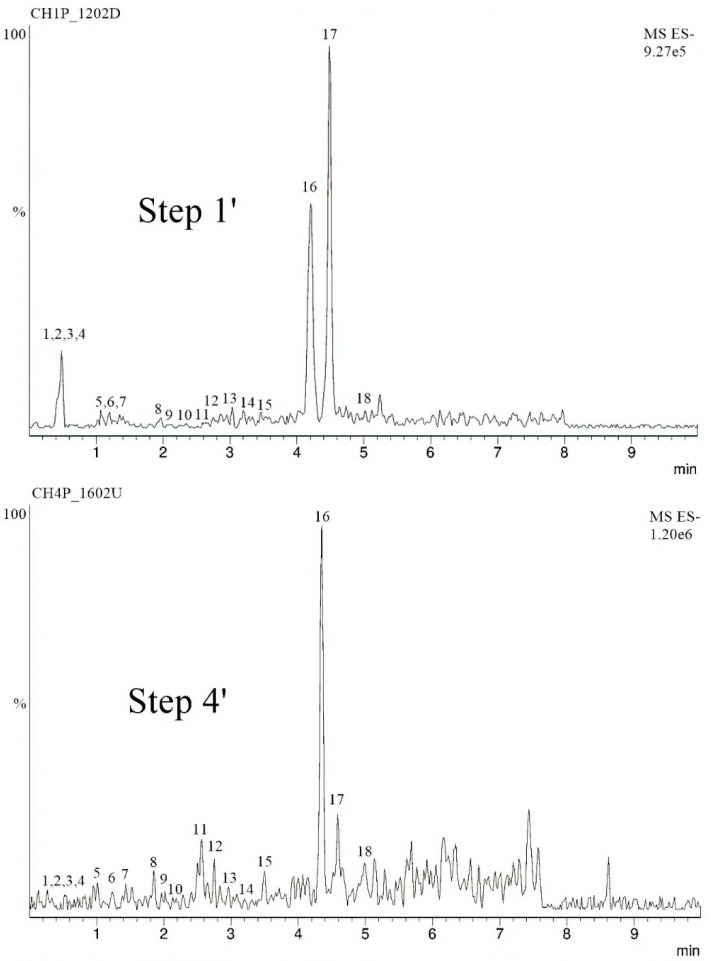
Base peak chromatograms comparing steps 1′ and 4′ of the seedling leaf explants from *Clidemia hirta.*

The analyses on *Clidemia hirta* and *Physalis angulata* leave explants (respectively in Figure 12 and Figure S3) show that the targeted metabolites chosen as markers are detected for both species in step 1′ and 4′, even if the overall chemical profile seems to be modified in *Clidemia hirta* between the two steps. These results are summarized in Table 3.

In conclusion, among the eight plant species chosen for their differences among one another, a majority of the reference marker compounds could still be found in leaf explants after being grown in culture meaning that the chemical diversities in these explants were not lost during the procedure allowing the transfer in vitro. In other words, it seems that we assessed enough plant material to hypothesize that if we start inducing callus from these tissues it will be possible to access an interesting chemodiversity as metabolic pathways seem to be still active. As a prospective for future work direction, the next step would be to characterize the diversity of metabolites produced by the callus, with similar chemical profile analyses based on mass spectrometry. If it is confirmed, it would mean that calli originating from plant species not very well characterized chemically could be used as a constant source of new or unexplored compounds. In this sense, in vitro tissue culture will allow gathering the necessary biomass to explore thoroughly the chemical diversity lying in natural products originating from plants, and several times if necessary.

As expected, establishing in vitro culture from any plant species harvested *in natura* can be challenging. But using the global approach summarized in Figure 4**,** our results show that a combination of alternatives could overcome the limitations due to the specificities of some plant species and that it is feasible to establish in vitro cultures for about two-thirds of the intended plant species, the last third failing due to harvest limitations and plant species characteristics.

Furthermore, we found in these leaf tissues in culture many of the compounds that make plant chemodiversity so special. In our view, the present work contributes to the development of alternative methodologies aiming at making available sustainable sources of plant secondary metabolites from plant species originating from all biodiversities, as exemplified here with the Amazonian biodiversity.

## 3. Materials and Methods

### 3.1. Plant Material and Harvest

The botanical material was collected in three different locations, at the UFPA campus, at the Embrapa Amazônia Oriental, both in Belém, and in the municipality of Vigia, in the state of Pará, Brazil. Samples from the following botanical species were collected: *Peperomia pellucida* (L.) Kunth [Piperaceae], *Calycophyllum spruceanum* (Benth.) Hook.f. ex K. Schum. [Rubiaceae], *Vatairea guianensis* Aubl. [Fabacae], *Swietenia macrophylla* King [Meliaceae], *Physalis angulata* L. [Solanaceae], *Phyllanthus brasiliensis* (Aubl.) Poir. [Phyllanthaceae], *Clidemia hirta* (L.) D. Don [Melastomataceae] and *Stryphnodendron pulcherrimum* (Willd.) Hochr. [Leguminosae]. Only the common Latin name of the plants will be used thereafter. Specimens have been deposited in the Herbarium of Embrapa Amazônia Oriental, whose voucher numbers are listed in Table 1 and access to genetic resources declared (Nagoya agreements). The fruits and seeds at the origin of axenic plantlets culture were collected on UFPA campus from the same specimens used for the voucher.

### 3.2. Chemicals

#### 3.2.1. General

UHPLC-grade methanol was purchased from SK Chemicals^®®^ (Pangyo-ro, South Korea) and acetonitrile (Lichrosolv^®®^) from Merck Millipore (Darmstadt, Germany). Formic acid (Lichrosolv^®®^ 98–100%) was also acquired from Merck Millipore. Absolute ethyl alcohol (99.5% Anidrol^®®^) was purchased from Soltech (Diadema, Brazil). Ultra-pure water was produced by a Milli-Q system.

#### 3.2.2. Standards

Standards of the compounds 12α-acetoxyl-20β,21β-22α,23α-diepoxyswietephragmin C, 12α-acetoxyswietephragmin D, 3β-*O*-detigloyl-3β-*O*-benzoyl-6-*O*-acetylswietephragmin D, 6-acetoxy-12α-deacetoxyl-8,9,30-ortho-tigloylate-swietemacrophine, 12α-acetoxyswietephragmin C, 8,9,30-*ortho*-tigloylate swietemacrophine, 3β-*O*-detigloyl-3β-*O*-benzoyl-6-*O*-acetylswietephragmin E, 3β-*O*-detigloyl-3β-*O*-benzoyl-12α-acetoxyswietephragmin D were previously isolated from *S. macrophylla* leaves [12,40,41] standards physalin B, physalin G, physalin D and isophysalin B were from *P*. *angulata* leaves [39] and arabelline, 4-*O*-β-d-apiofuranosyl-(1′′′,6′′)-β-D-glucopyranosyl-diphyllin, 5-*O*-β-D-glucopyranosyljusticidin B, cleistanthin B, phyllanthostatin A, tuberculatin and justicidin B from the leaves of *P. brasiliensis* [48].

### 3.3. In Vitro Cultures

#### 3.3.1. Establishment of Cultures from Leaves Collected in Natura

Fresh leaf material was harvested in natura and treated following the different steps illustrated in Figure 1. Depending on the size of the leaf/leaflet, 50 to 80 of them were harvested and pre-cleaned individually with water and soap and thoroughly rinsed. They were then placed in a jar of a biocidal solution containing the fungicide Dithane NT^®®^ (mancozeb 80% m/m) at 2.5 g/L commercial product and tetracycline at 80 mg/L with several drops of Tween 20 during 4 h for herbaceous and shrubs species or 5 h for tree species. Following these cleaning steps (Figure 1), the leaves were taken to a sterile environment (laminar airflow cabinet) and placed into sterile water to complete two separate washes and then into sterile polyvinylpyrrolidone solution (PVP) at 1 g/L for two further separate washes. They were then placed in EtOH (70%) during 5 and 30 s, respectively for herbaceous and shrubs, and for tree species and then, during 20 and 30 min, in commercial sodium hypochlorite solution (2–2.5% active compound) with several drops of Tween 20. Following this surface sterilization process, the leaves were washed separately with PVP sterile solution and sterile water (twice). Explants of around 0.5–1 cm^2^ were cut and 120 of them placed into ~40 mL of Murashige and Skoog (MS) [71] liquid medium diluted twice (MS/2) and supplemented by activated charcoal (2.5 g/L), ascorbic acid (300 mg/L) and PVP (1 g/L). They were incubated in darkness at 24 °C (± 1 °C) for 3 days. From the 120 initial explants, those that remained free of visible contamination after this period were moved to solid MS media supplemented with ascorbic acid (100 mg/L) and phytagel (2.5 g/L) and incubated at 25 °C (± 1 °C) with a 12 h/12 h dark photoperiod supplied by Q315F photoperiod incubator (Quimis, São Paulo, Brazil) with an illuminance of ~2000 Lux.

After six days of culture, the impact of the different steps of the establishment of in vitro cultures was evaluated through two different parameters: efficiency of the surface sterilization and impact of the procedure on the tissues. For the efficiency of the surface sterilization, explants were checked daily and the ones with visible microbial contamination were discarded. As a result, a decontamination rate (number of contaminant-free explants/initial number of explants × 100) was calculated after six days of culture on the batch of 120 initial explants for each procedure. The experiment was repeated three times for each plant species and the corresponding mean values and standard deviations were calculated.

Regarding the impact of the procedure on tissues after the different steps of surface sterilization, it was evaluated after six days of culture through the observation and ranking of the visual aspect of the explant tissues. The rational scale that was used allowed to reduce subjectivity and to standardize the evaluation between the different operators and from one repetition or species to another. Each one of the contamination-free explants was evaluated. They were scored in function of the intensity and size of the explants area showing a change in the color and texture of the explant and the presence of oxidative browning. The scale run from 5 to 0 and the scores were attributed individually as follows: score 5 (less than 10% of the explant area was visibly affected); score 4 (around one-third of the explant area was visibly affected); score 3 (around one half of the explant area was visibly affected); score 2 (around two-thirds of the explant area were visibly affected); score 1 (around 10% of the area does not seem affected). 0.5 (all the explant area was affected); 0 (all the explant area was affected, and exudation of oxidation browning compounds was visible in the culture media). An average score was determined on the evaluation of the batch of contamination-free explants. The experiment was realized three times for each plant species and the corresponding mean values and standard deviations were calculated. Furthermore, biological samples (8 to 12 explants) were collected after steps 1, 2, 3, and 4 of the previous procedure (Figure 1) for the phytochemical analyses. They were kept frozen until drying and extraction.

#### 3.3.2. Establishment of Axenic Plantlets Cultures Originating from Seeds Collected in Natura

Because working with some species can lead to leaf explants with low viability and thus not usable for tissue culture, we used an alternative route: growing axenic plantlets in vitro from which leaves can also be excised to explants. The winged tegument from seeds of *Swietenia macrophylla* was removed, and the seeds rehydrated during 24 h. Afterward, they were immersed during 30 min in the biocide solution described above, washed three times and placed into hypochlorite solution (2–2.5% active compound) with several drops of Tween 20 for 10 min. Finally, they were washed three times again with sterile water and placed onto a solid MS medium. Fruits from *Clidemia hirta* and *Physalis angulata* were directly immersed into hypochlorite solution (2–2.5% a.c.) with several drops of Tween 20 for 15 min, washed with sterile water three times and cut open in sterile conditions to take the seeds out. Seeds of *Stryphnodendron pulcherrimum* followed the same treatment after 1h rehydration. Seeds were then placed onto solid MS medium for *Physalis angulata* and *Stryphnodendron pulcherrimum* and solid MS medium diluted four times for *Clidemia hirta*. All seeds were incubated at 27 °C (± 1 °C) with 12 h/12 h dark photoperiod supplied by daylight and germination and development of axenic plantlets registered along with possible contaminations. Germination experiments were repeated three times except for *Stryphnodendron pulcherrimum* due to the low availability of seeds.

### 3.4. Phytochemical Analyses

The biological material that had been collected was kept frozen at −20 °C until submitted to dryness in an air-forced oven at 45 °C. Then, the appropriate mass was grounded in a mortar with a pestle and subjected to extraction.

#### 3.4.1. Extraction Procedure and Sample Preparation for Secondary Metabolite Analysis

During the extraction procedures, many tests were performed for each plant material to set the conditions able to extract the biomarkers compounds efficiently. Using an ultrasonic bath, the type of solvents or mixtures thereof, time of extraction, and liquid-to-solid ratio were modified in order to obtain the best conditions for each species. After the extraction, each extract was suspended at the proportion of 1mg/mL of solvent and cleaned up on SPE C18 cartridges (Strata-C18-E, 50 mg, 1 mL, Phenomenex, Torrance, CA, USA) previously conditioned with 1 mL ACN and subsequently 1 mL of water. Then, the cartridge was loaded with 1 mL of sample extract that was eluted twice with water/ACN (20:80), total volume being 1 mL.

For each species collected, a sample of plant material was taken before any treatment (referred to as step 0), and analyzed by LC-MS system (Xevo G2-SToF from Waters corp., Milford, MA, USA) to be used as a reference for each species. At this initial analytic step, some chemical constituents were identified and used as biomarkers for the present work. All samples were prepared and analyzed in triplicate. Aliquots of the treated samples were solubilized at a concentration of 1 mg/mL in water: ACN (20:80), filtered through a syringe filter (0.22 µm) and injected in an LC-MS instrument (5 µL).

#### 3.4.2. LC-MS Analyses

Chromatographic fingerprinting of the extracts was performed on a LC system coupled to an ESI-QTof mass spectrometer XEVO G2-S QTof (Waters, Milford, MA, USA) with electrospray ionisation (ESI) in negative and positive ion modes. The separation was achieved on a BEH RP18 column (50 mm, 2.1 mm, 1.7 μm) (Waters) at a flow rate of 0.3 mL/min. The solvent system used was a mixture of water with (A) and ACN (B) in a linear gradient mode from 5 to 95% B in 10 or 20 min, according to method development to each plant. The capillary voltage was set to 3 KV and the capillary temperature to 150 °C. Data acquisition was performed at the range 50–1200 *m*/*z* ratio, except for *Clidemia hirta* that was established for a range of 50–2000 *m*/*z* ratio. In the figures, the base peak ion chromatograms (BPI) extracted from total ion chromatograms (TIC) are presented in order to reduce the background noise and show the ions responsible for the signal. The identification of the compounds was done using the molecular masses, together with the fragmentation patterns, spectra and compared with data from the literature, in particular from da Costa et al. [72] and Li et al. [73].

## Figures and Tables

**Table 1:** List of selected species.

Species Name [Botanical Family]	Herbarium Voucher Number	Type of Plant	Class of Compounds of Interest	Parts of the Plant Accumulating the Compounds	Ref
*Physalis angulata* L. [Solanaceae]	IAN 197200	Herbaceous	Physalins (terpenoids)	Stems, leaves, fruits	[39]
*Swietenia macrophylla* King [Meliaceae]	IAN 197201	Tree species	Limonoids	Leaves	[12,40,41]
*Clidemia hirta* (L.) D. Don [Melastomatacea]	IAN 197199	Shrub	Tannins	Leaves	[42]
*Calycophyllum spruceanum* (Benth.) Hook.f. ex K. Schum. [Rubiaceae]	IAN 188633	Tree species	Triterpenes	Leaves	NP
*Vatairea guianensis* Aubl. [Fabaceae]	IAN 187050	Tree species	Isoflavonoids	Leaves and stems	[43,44,45]
*Peperomia pellucida* (L.) Kunth [Piperaceae]	IAN 197198	Herbaceous	ArC_2_ dimers, flavonoids and bisnorlignans	Whole plant	[46,47]
*Phyllanthus brasiliensis* (Aubl.) Poir. [Phyllanthaceae]	IAN 185501	Shrub	Flavonoids and lignans	Leaves	[48]
*Stryphnodendron pulcherrimum* (Willd.) Hochr. [Fabaceae]	IAN 199608	Tree species	Tannins and Flavonoids	Stem bark and leaves	NP

NP: unpublished.

**Table 2:** Survey of some selected compound markers presence during the different steps of in vitro cultures establishment.

Plant Species	Number of Compound Markers Which Presence Has Been Validated in Leaf Samples Collected “*In Natura*” (Step 0)	Proportion of Markers Detected After Pre-Cleaning (Step 1)	Proportion of Markers Detected After Cleaning of the Leaves (Steps 1–2)	Proportion of Markers Detected After Cleaning and Surface Sterilization (Steps 1–3)	Proportion of Markers Detected after 6 Days of Culture (Steps 1–4)
*Physalis angulata*	18 (8 physalins and 10 unknown compounds)	18/18	18/18	18/18	18/18
*Swietenia macrophylla*	10 limonoids	10/10	10/10	10/10	10/10
*Clidemia hirta*	18 (2 phenolic acids, 2 glycosylated flavonoids, 5 ellagitannins and 8 unknown compounds)	18/18	18/18	18/18	17/18
*Calycophyllum spruceanum*	12 (2 phenolic acids, 4 flavonoids and 6 unknown compounds)	12/12	12/12	12/12	12/12
*Vatairea guianensis*	15 (3 flavones, 8 isoflavones and 4 unknown compounds)	15/15	15/15	15/15	ND
*Peperomia pellucida*	09 (1 flavanoid, 1 lignan, 3 coumarins, 2 lactones and 4 ArC_2_ dimers)	09/09	09/09	09/09	05/09
*Phyllanthus brasiliensis*	14 (7 flavonoids and 7 lignans)	14/14	14/14	07/14	07/1
*Stryphnodendron pulcherrimum*	27 (11 tannins, 3 flavonoids and 13 unknown compounds)	27/27	14/27	10/27	07/27

ND: not determined (no contaminant-free explants available).

**Table 3:** Survey of the detection of the selected compound markers when the starting material is leaves from axenic plantlets.

Plant Species	Number of Compound Markers Selected from Mature Leaf Samples Collected “*In Natura*”	Proportion of Markers Detected in the Leaves of Young Axenic Plantlets (Step 1′)	Proportion of Markers Detected in Leaf Explants from the Axenic Plantlets after 6 Days of Culture (Step 4′)
*Swietenia macrophylla*	10	10/10	10/10
*Clidemia hirta*	18	18/18	17/18
*Physalis angulata*	18	18/18	18/18

## Supplementary Materials

The following are available online at https://www.mdpi.com/1420-3049/25/24/5992/s1, Figure S1: Base peak ion chromatograms comparing steps 1 to 3 of the leaf explants from *Vatairea guianensis*; Figure S2: Base peak ion chromatograms comparing steps 1 to 4 of the leaf explants from *Peperomia pellucida*; Figure S3: Base peak ion chromatograms comparing steps 1′ and 4′ of the seedling leaf explants from *Physalis angulate*; Figure S4: Base peak ion chromatograms (ES+) of *Physalis angulata* with several mass spectra extracted for peaks of interest; Figure S5: Base peak ion chromatograms (ES-) of *Clidemia hirta* with several mass spectra extracted for interesting peaks; Figure S7: Base peak ion chromatograms of *Calycophyllum spruceanum* with several mass; Figure S8: Base peak ion chromatograms (ES+) of *Vatairea guianensis* with several mass spectra extracted for interesting peaks; Figure S9: Base peak ion chromatograms (ES+) *Peperomia pellucida* with several mass spectra extracted for interesting peaks; Figure S10: Base peak ion chromatograms (ES- and ES+) of *Phyllantus brasiliensis* with several mass spectra extracted for interesting peaks; Figure S11: Base peak ion chromatograms (ES-) of *Stryphnodendron pulcherrimum* with several mass spectra extracted for interesting peaks; Table S1: Characterization of chemical constituents of *Physalis angulata* detected on plants leaves by UPLC−ESI−QToF−MS, Table S2: Characterization of chemical constituents of *Swietenia macrophylla* detected on plants leaves by UPLC−ESI−QToF−MS, Table S3: Characterization of chemical constituents of *Clidemia hirta* detected on plants leaves by UPLC−ESI−QToF−MS, Table S4: Characterization of chemical constituents of *Calycophyllum spruceanum* detected on plants leaves by UPLC−ESI−QToF−MS, Table S5: Characterization of chemical constituents of *Vatairea guianensis* detected on plants leaves by UPLC−ESI−QToF−MS, Table S6: Characterization of chemical constituents of *Peperomia pellucida* detected on plants leaves by UPLC−ESI−QToF−MS, Table S7: Characterization of chemical constituents of *Phyllantus brasiliensis* detected on plants leaves by UPLC−ESI−QToF−MS, Table S8: Characterization of chemical constituents of *Stryphnodendron pulcherrimum* detected on plants leaves by UPLC−ESI−QToF−MS. Scheme S1. Isotope standard for peak 1 of *Peperomia pellucida*, calculations of molecular formula and error of mass ppm (Software Masslynx 4.1); Scheme S2. Isotope standard for peak 3 of *Peperomia pellucida*, calculations of molecular formula and error of mass ppm (Software Masslynx 4.1); Scheme S3. Isotope standard for peak 4 of *Peperomia pellucida*, calculations of molecular formula and error of mass ppm (Software Masslynx 4.1); Scheme S4. Example (step 1: insert spectrum experimental) for peak 2 of *Peperomia pellucida*, calculations of similarity MS/MS in MoNA (https://mona.fiehnlab.ucdavis.edu/); Scheme S5. Example (step 2: similarity result) for peak 2 of *Peperomia pellucida*, calculations of similarity MS/MS in MoNA (https://mona.fiehnlab.ucdavis.edu/); Scheme S6. Example (step 3: match of spectrum database and experimental) for peak 2 of *Peperomia pellucida*, calculations of similarity MS/MS in MoNA (https://mona.fiehnlab.ucdavis.edu/).
