# Supplementary material for: Secure and Sustainable Sourcing of Plant Tissues for the Exhaustive Exploration of Their Chemodiversity
Source: Molecules. 2020 Dec 18;25(24):5992. doi: 10.3390/molecules25245992 (PMC7766005; doi:10.3390/molecules25245992)

Secure and sustainable sourcing of plant tissues for the exhaustive exploration of their chemodiversity

Rhodin C. Joseph[1], Matheus Silva da Fonseca Diniz[1], Viviane Magno do Nascimento[1], Abraão de Jesus Barbosa Muribeca[2], Johan Carlos Costa Santiago[2], Luziane da Cunha Borges[2], Paulo Roberto da Costa Sá[2], Paulo Wender Portal Gomes[2], Júlio César da Silva Cardoso[3], Marcela Natalia Rocha de Castro[4], Thais Fiusa[5], Hervé Rogez[1], Sylvain Darnet[1], Mara Silvia Pinheiro Arruda[2], Milton Nascimento da Silva[2,*], Alberto Cardoso Arruda[3], Jean A. Boutin[6], Consuelo Yumiko Yoshioka e Silva[4], Emmanuelle Lautié[1*]

[1] Centre for Valorization of Amazonian Bioactive Compounds (CVACBA), Federal University of Pará (UFPA), Espaço Inovação, Av. Perimetral da Ciência, 66.095-630 Belém, Pará, Brazil.
[2] Chemistry Post-Graduation Program, Institute of Exact and Natural Sciences, UFPA, Av. Bernardo Sayão, 66.075-110 Belém, Pará, Brazil.
[3] School of Chemistry, Institute of Exact and Natural Sciences, UFPA, Av. Bernardo Sayão, 66.075-110 Belém, Pará, Brazil.
[4] Pharmaceutical Science Post-Graduation Program, Faculty of Pharmacy, UFPA, Av. Bernardo Sayão, 66.075-110 Belém, Pará, Brazil.
[5] Laboratórios Servier do Brasil, Estrada dos Bandeirantes, 4211, Rio De Janeiro, RJ 22775-113 Brazil
[6] Institut de Recherches Internationales Servier, 50 rue Carnot, 92884 -Suresnes Cedex, France.
* Correspondence: elautie.dias@gmail.com

Supplementary Material

A. <u>Supplementary Figures:</u>

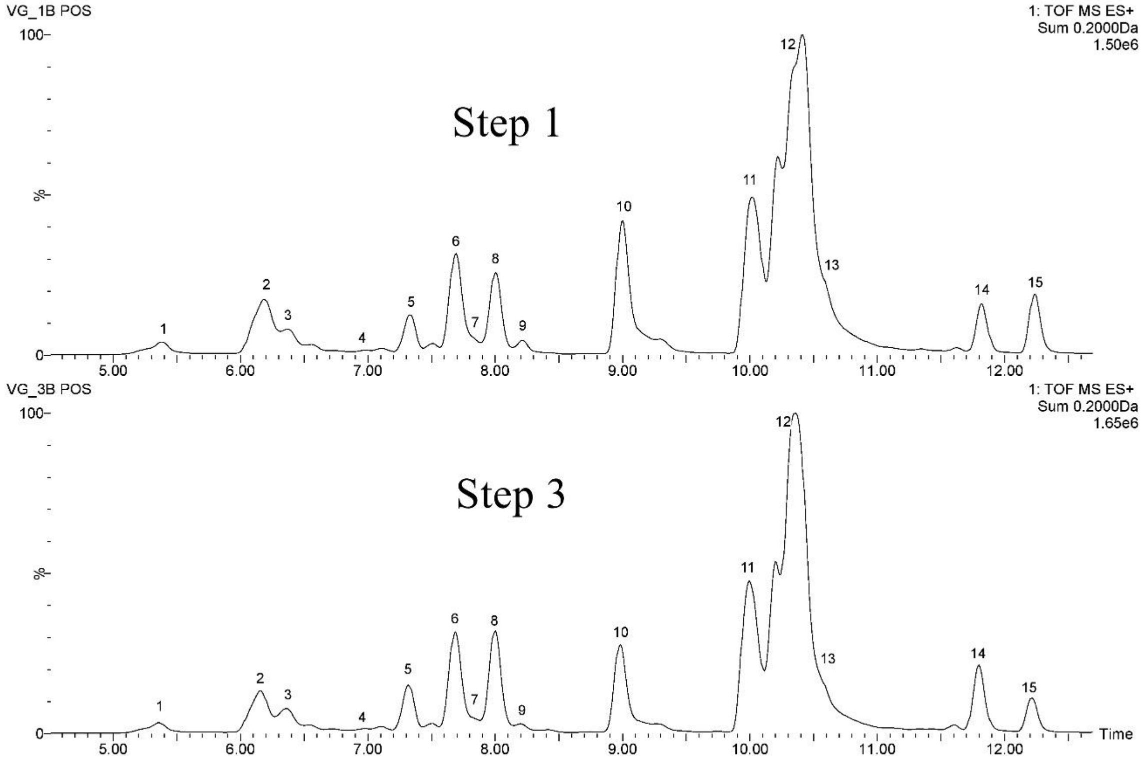

Figure S1. Base peak ion chromatograms comparing steps 1 to 3 of the leaf explants from *Vatairea guianensis.*

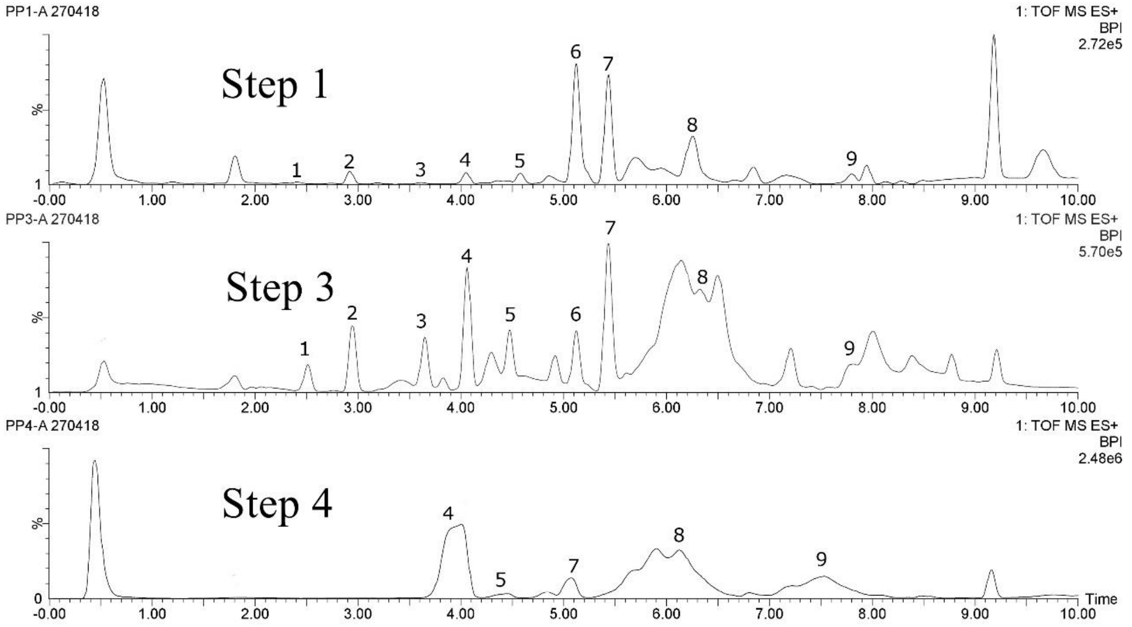

Figure S2. : Base peak ion chromatograms comparing steps 1 to 4 of the leaf explants from *Peperomia pellucida*.

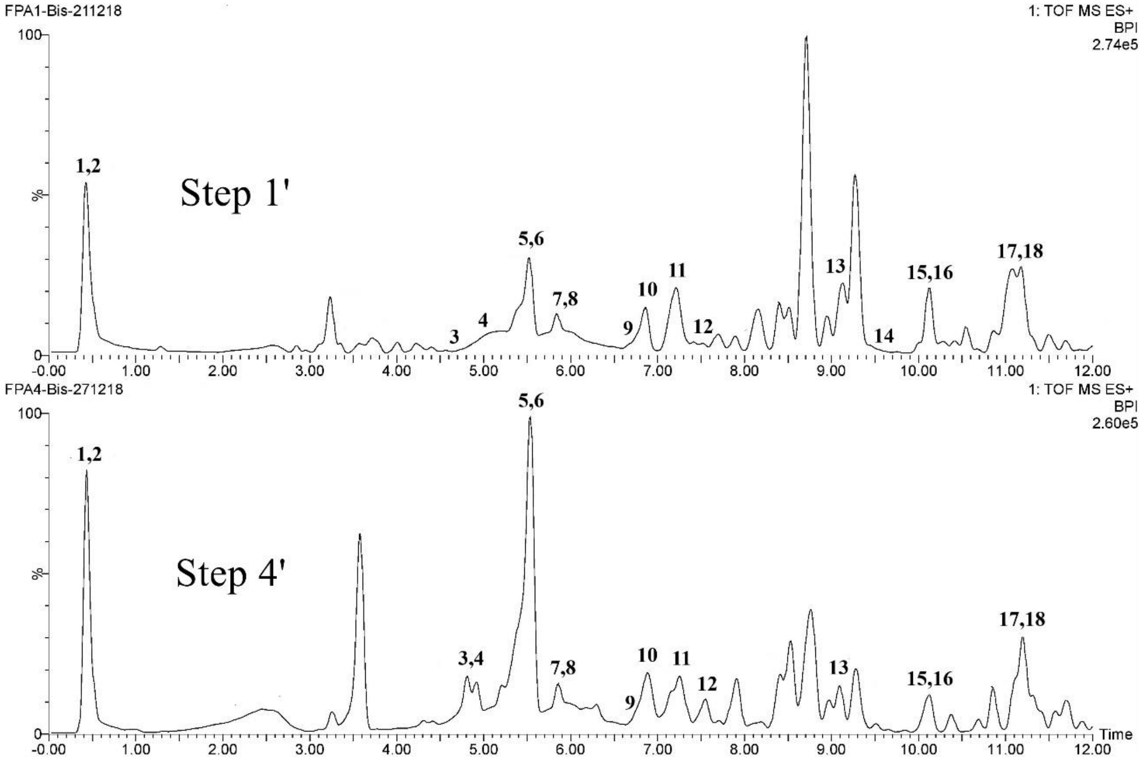

Figure S3: Base peak ion chromatograms comparing steps 1′ and 4′ of the seedling leaf explants from *Physalis angulata*.

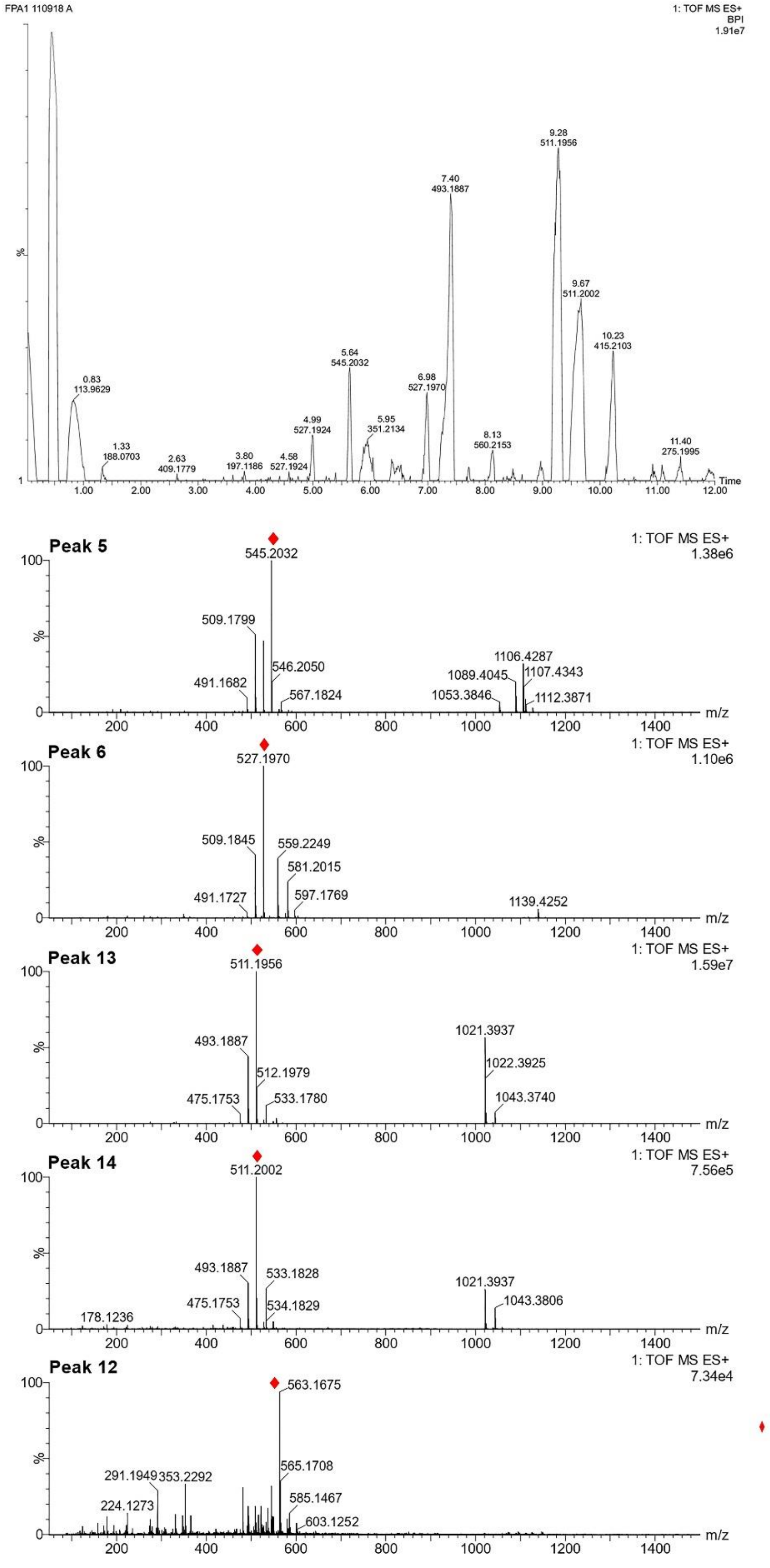

Figure S4. Base peak ion chromatograms (ES+) of Physalis angulata with several mass spectra extracted for peaks of interest.

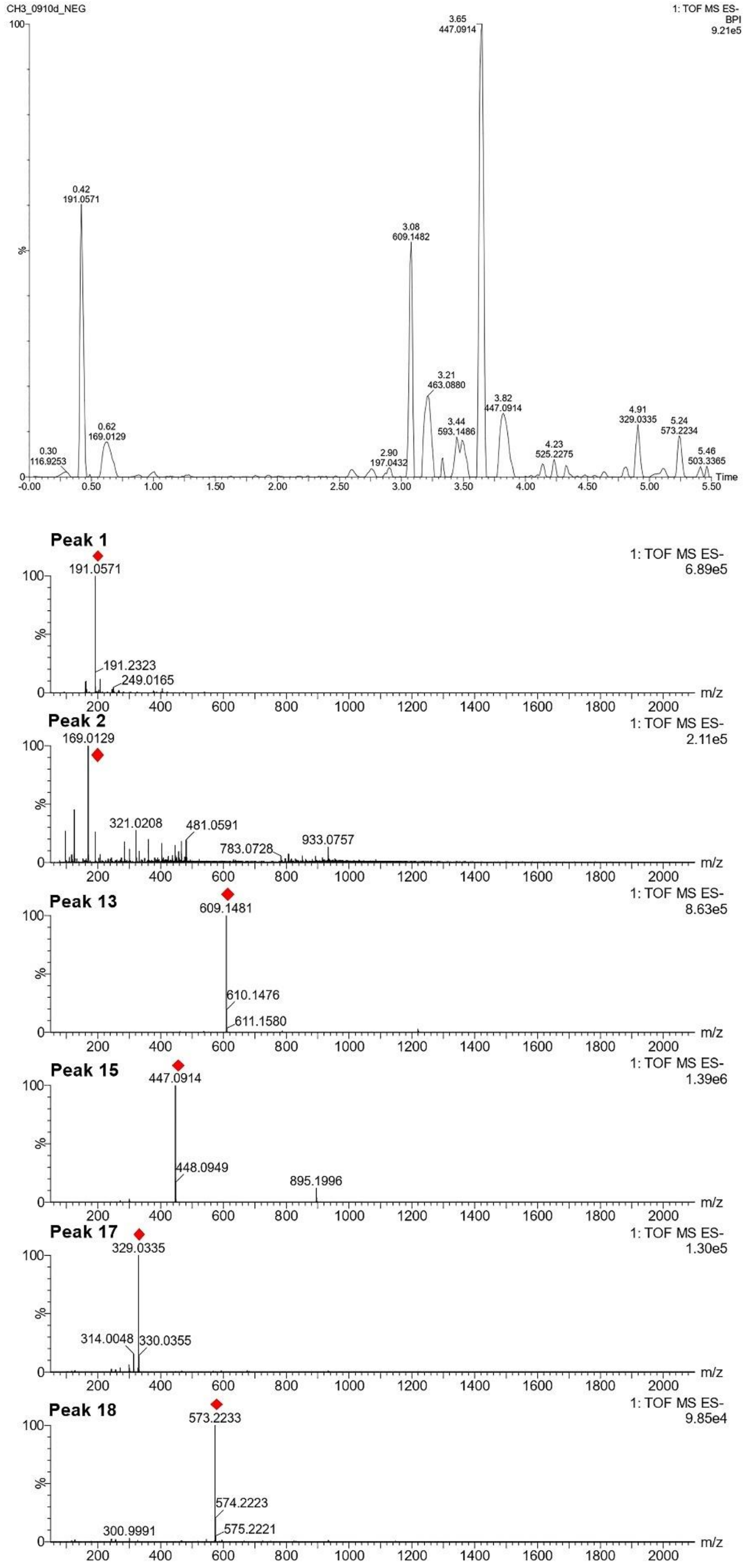

Figure S5. Base peak ion chromatograms (ES-) of *Clidemia hirta* with several mass spectra extracted for interesting peaks.

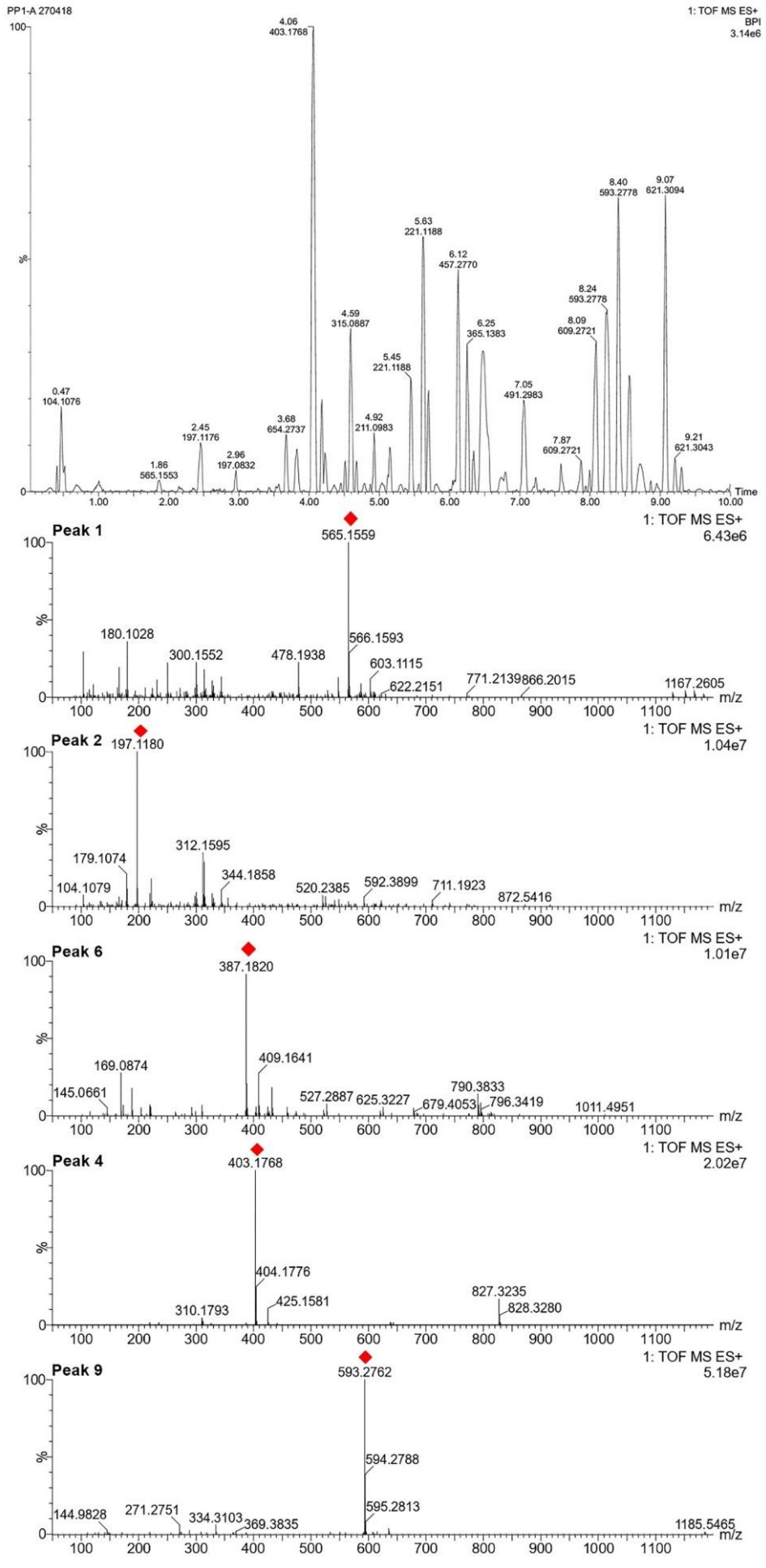

Figure S7. Base peak ion chromatograms of *Calycophyllum spruceanum* with several mass spectra extracted for interesting peaks.

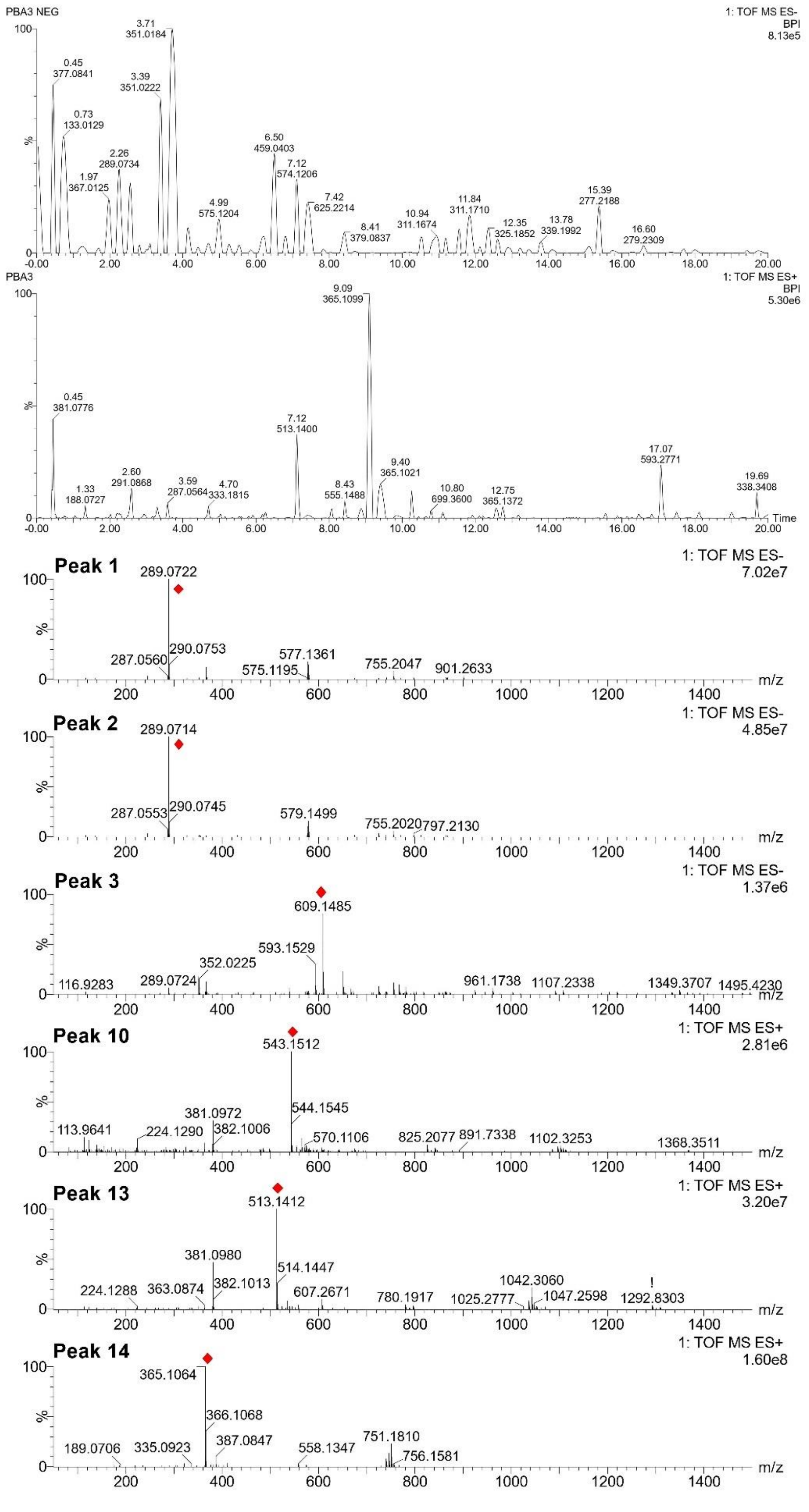

Figure S8. Base peak ion chromatograms (ES$^+$) of *Vatairea guianensis* with several mass spectra extracted for interesting peaks.

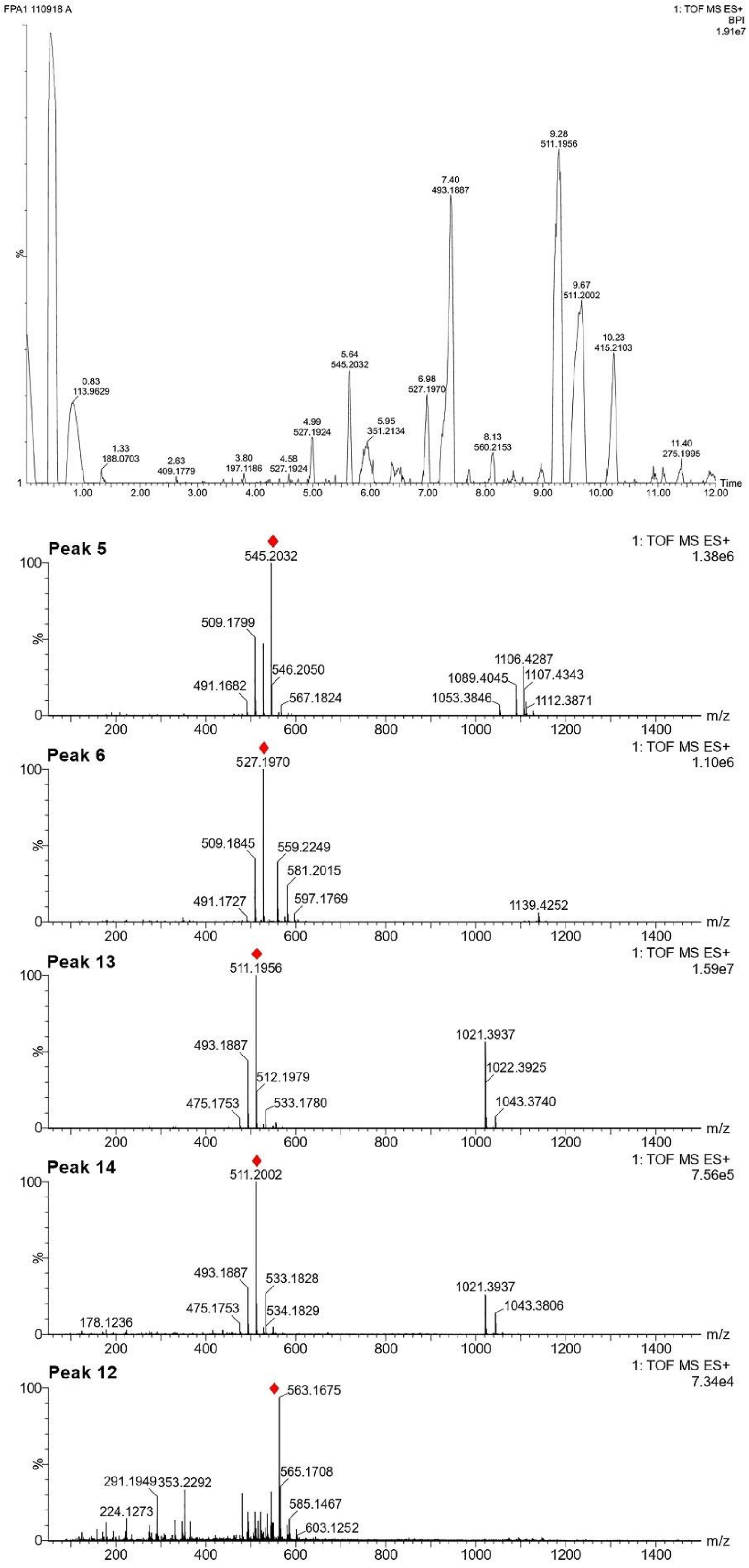

Figure S9. Base peak ion chromatograms (ES+) *Peperomia pellucida* with several mass spectra extracted for interesting peaks.

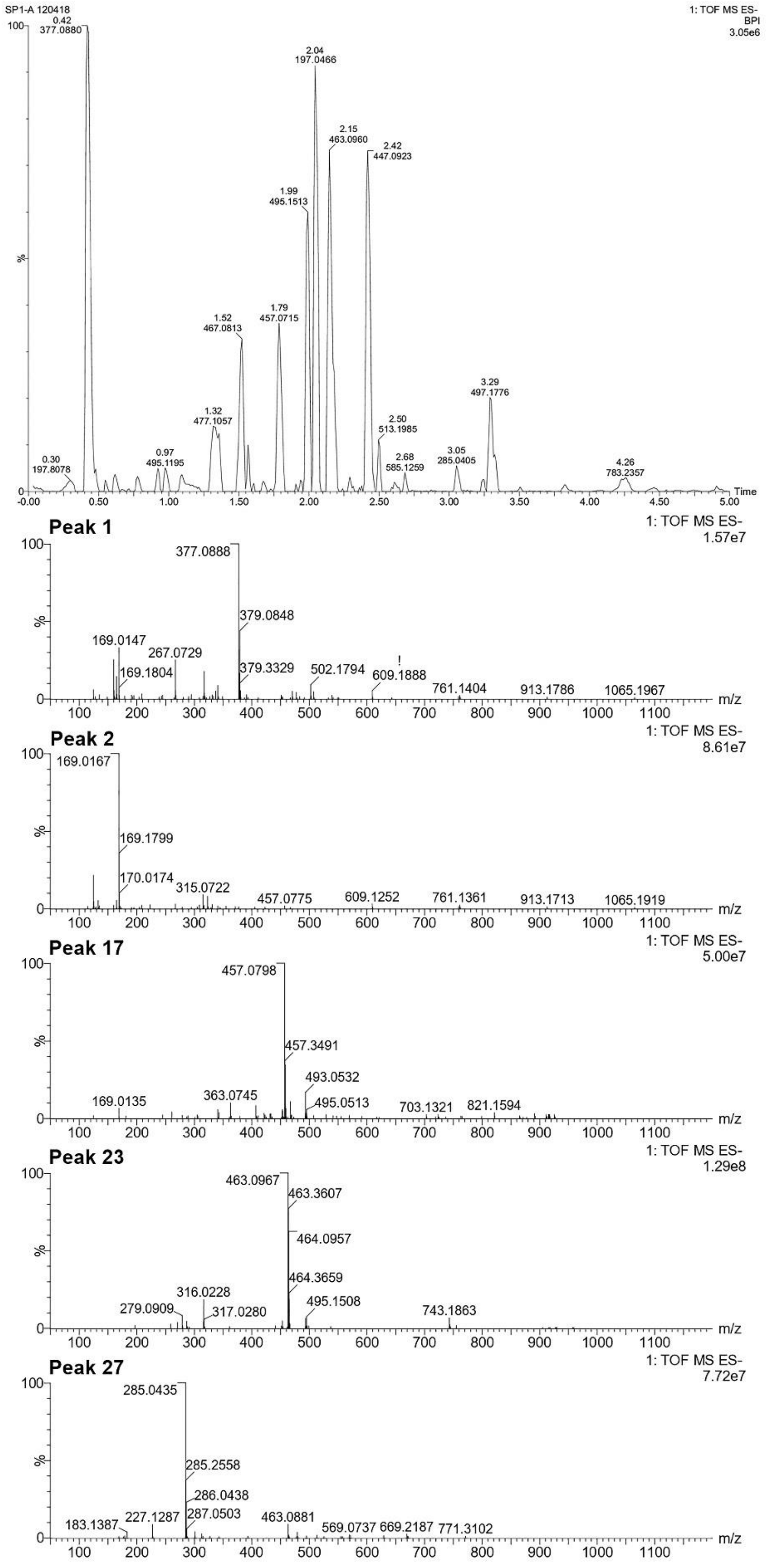

Figure S10. : Base peak ion chromatograms (ES^{-} and ES^{+}) of *Phyllantus brasiliensis* with several mass spectra extracted for interesting peaks.

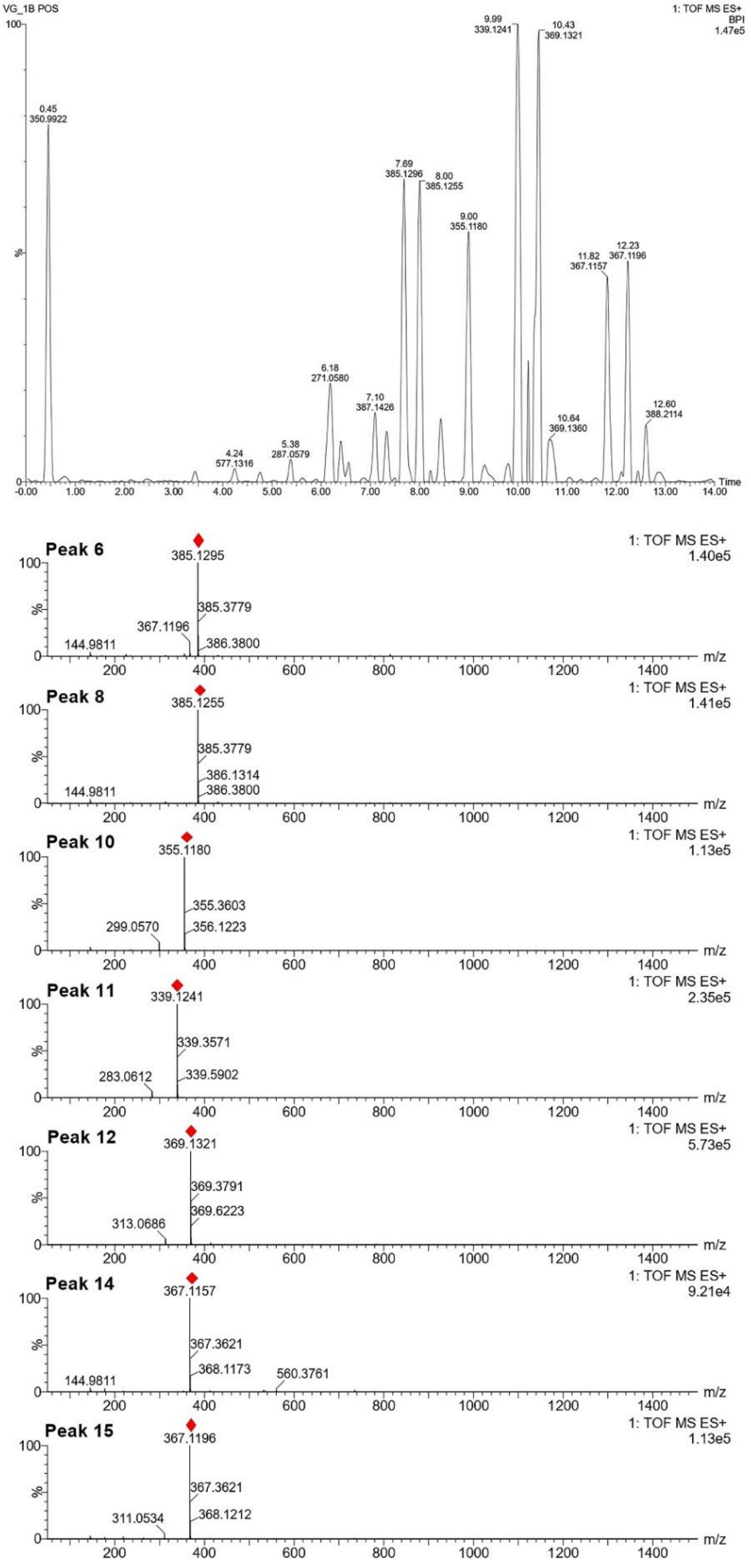

Figure S11. Base peak ion chromatograms (ES$^-$) of *Stryphnodendron pulcherrimum* with several mass spectra extracted for interesting peaks.

B. Supplementary Tables:

Table S1. Characterization of chemical constituents of *Physalis angulata* detected on plants leaves by UPLC−ESI−QToF−MS.

Peak	*tR*/min	$[M+H]^+$ (mass error, ppm)	Molecular formula	Proposed compound	Ref or database
**1**	0.44	455.1116	-	Unknown	-
**2**	0.50	453.2090	-	Unknown	-
**3**	4.65	509.1805	-	Unknown	-
**4**	4.98	527.1932 (2.84)	$C_{28}H_{30}O_{10}$	Unknown	-
**5**	5.62	545.2045 (4.03)	$C_{28}H_{32}O_{11}$	Physalin D	Standard
**6**	5.63	527.1932 (2.84)	$C_{28}H_{30}O_{10}$	Physalin G	Standard
**7**	6.12	351.2169	-	Unknown	
**8**	6.21	351.2130	-	Unknown	
**9**	6.67	351.2169	-	Unknown	
**10**	6.97	527.1979 (2.84)	$C_{28}H_{30}O_{10}$	Unknown	-
**11**	7.42	527.1932 (2.84)	$C_{28}H_{30}O_{10}$	Unknown	-
**12**	7.56	563.1695	$C_{28}H_{31}ClO_{10}$	Physalin H	-
**13**	9.26	511.1998 (5.86)	$C_{28}H_{30}O_{9}$	Physalin B	Standard
**14**	9.65	511.1998 (5.86)	$C_{28}H_{30}O_{9}$	Isophysalin B	Standard
**15**	10.30	351.2551	-	Unknown	
**16**	10.46	351.2551	-	Unknown	
**17**	10.93	553.4257	-	Unknown	
**18**	11.08	553.4209	-	Unknown	

Table S2. Characterization of chemical constituents of *Swietenia macrophylla* detected on plants leaves by UPLC−ESI−QToF−MS.

**Peak**	***tR*/min**	**[M+H]$^+$ (mass error, ppm)**	**Molecular formula**	**Proposed compound**	**Ref or database**
**1**	6.18	773.3000 (2.71)	$C_{39}H_{48}O_{16}$	12α-acetoxyl-20β,21β-22α,23α-diepoxyswietephragmin C	Standard
**2**	6.37	749.2811 (0.26)	$C_{40}H_{44}O_{14}$	3β-O-detigloyl-3β-O-benzoyl-6-O-acetylswietephragmin D	Standard
**3**	6.48	739.2936 (4.05)	$C_{39}H_{46}O_{14}$	6-acetoxyl-12α-deacetoxyl-8,9,30-ortho-tigloylate-swietemacrophine	Standard
**4**	6.93	739.2936 (4.05)	$C_{39}H_{46}O_{14}$	8,9,30-ortho-tigloylate-swietemacrophine.	Standard
**5**	7.25	749.2811 (0.26)	$C_{40}H_{44}O_{14}$	12α-acetoxy-swietephragmin C	Standard
**6**	7.25	727.2968 (0.27)	$C_{38}H_{46}O_{14}$	12α-acetoxyswietephragmin D	Standard
**7**	7.28	741.3146 (3.23)	$C_{39}H_{48}O_{14}$	3β-O-detigloyl-3β-O-benzoyl-12α-acetoxyswietephragmin D	Standard
**8**	7.28	741.3146 (3.27)	$C_{39}H_{48}O_{14}$	6-O-acetylswietephragmin E	Standard
**9**	7.66	763.2941 (3.27)	$C_{41}H_{46}O_{14}$	3β-O-detigloyl-3β-O-benzoyl-6-O-acetylswietephragmin E	Standard
**10**	7.87	763.2941 (3.27)	$C_{41}H_{46}O_{14}$	3β-O- detigloyl-3 β-O-benzoyl-12α-acetoxy-swietephragmin C	Standard

Table S3. Characterization of chemical constituents of *Clidemia hirta* detected on plants leaves by UPLC−ESI−QToF−MS.

Peak	*tR*/min	$[M-H]^-$ (mass error, ppm)	Molecular formula	Proposed compound	Ref or database
**1**	0.42	191.0571 (7.85)	$C_7H_{12}O_6$	Quinic Acid	MONA ID 206290<br>Kumar el al., 2015[45]<br>Bouhafsoun et al., 2018[46]<br>Engström, Pälijärve Salminen, 2015[47]
**2**	0.46	169.0129 (4.73)	$C_7H_6O_5$	Gallic Acid	MONA ID 8162
**3**	0.52	300.9991 (2.32)	$C_{14}H_6O_6$	Ellagic Acid	METLIN ID 3430
**4**	052	933.0632 (0.21)	$C_{41}H_{26}O_{26}$	Ellagitannin a*	Bowers et al., 2018[48]<br>Abdellaoui et al., 2014[49]
**5**	1.01	933.0638 (0.21)	$C_{41}H_{26}O_{26}$	Ellagitannin b*	Bowers et al., 2018[48]<br>Abdellaoui et al., 2014[49]
**6**	1.15	783.0728 (6.00)	$C_{34}H_{24}O_{22}$	Ellagitannin c#	Bowers et al., 2018[48]<br>Abdellaoui et al., 2014[49]
**7**	1.28	933.0638 (0.42)	$C_{41}H_{26}O_{26}$	Ellagitannin d*	Bowers et al., 2018[48]<br>Abdellaoui et al., 2014[49]
**8**	2.18	977.0844 (5.32)	$C_{43}H_{30}O_{27}$	Unknown	-
**9**	2.37	807.0642 (4.83)	$C_{36}H_{24}O_{22}$	Ellagitannin e&	Bowers et al., 2018[48]<br>Abdellaoui et al., 2014[49]
**10**	2.60	635.0914 (4.72)	$C_{27}H_{24}O_{18}$	Unknown	-
**11**	2.77	461.0752 (5.85)	$C_{14}H_{22}O_{17}$	Unknown	-
**12**	2.95	197.0432 (9.13)	$C_9H_{10}O_5$	Unknown	-
**13**	3.08	609.1482 (4.43)	$C_{27}H_{30}O_{16}$	Rutin	METLIN ID 3677<br>GNPS ID 4679290<br>MONA ID 216
**14**	3.24	937.0892 (5.86)	$C_{41}H_{30}O_{26}$	Unknown	-
**15**	3.65	447.0914 (2.90)	$C_{21}H_{20}O_{11}$	Quercitrin	METLIN ID 43747<br>GNPS ID 4679290<br>GNPS ID 4679288<br>MONA ID 889<br>MONA ID 4679288

**16**	4.81	301.0346 (0.66)	$C_{15}H_{10}O_7$	Unknown	-
**17**	4.91	329.0335 (11.54)	$C_{16}H_{10}O_8$	Unknown	-
**18**	5.24	573.2233 (8.72)	$C_{26}H_{38}O_{14}$	Unknown	-

*$C_{41}H_{26}O_{26}$ isomers: ellagitannins called here as ***a***, ***b*** and ***d***; #$C_{34}H_{24}O_{22}$ an ellagitannin isomer called here as ***c***; &$C_{36}H_{24}O_{22}$: an ellagitannin called here as ***e***.

Table S4. Characterization of chemical constituents of *Calycophyllum spruceanum* detected on plants leaves by UPLC−ESI−QToF−MS

Peak	*tR*/min	$[M-H]^-$ (mass error, ppm)	Molecular formula	Proposed compound	Ref or database
**1**	0.42	191.0558 (1.04)	$C_7H_{12}O_6$	Quinic acid	MONA ID 206290<br>Kumar et al., 2017[32]
**2**	1.25	353.0872 (0.28)	$C_{16}H_{18}O_9$	Caffeoylquinic acid	MONA ID 78885<br>Willems et al., 2016[33]
**3**	1.75	577.1345 (0.17)	$C_{30}H_{26}O_{12}$	(epi) catechin dimer	HMDB ID 33973<br>Lv et al., 2015[34]
**4**	2.02	403.1240 (1.24)	$C_{17}H_{24}O_{11}$	Unknown	-
**5**	2.10	553.1381 (4.33)	$C_{21}H_{30}O_{17}$	Unknown	-
**6**	2.38	807.2630 (1.48)	$C_{25}H_{24}O_7$	Unknown	-
**7**	2.61	435.1463 (4.36)	$C_{25}H_{24}O_7$	Unknown	-
**8**	3.01	1027.2421 (8.46)	$C_{51}H_{48}O_{23}$	(epi) catechin trimer Monoglycoside	Rodrigues et al., 2007[35]
**9**	3.15	595.1282 (2.85)	$C_{26}H_{28}O_{16}$	Quercetin 3-O-α-arabinopyranosyl (1→6) β-glucopyranoside	Krenn et al., 2003[36]
**10**	3.58	579.1354 (0.69)	$C_{26}H_{28}O_{15}$	Kaempferol 3-O-β-D-glucopyranosyl (1→2) β-D-xylopyranoside	Yang et al., 2014[37]
**11**	4.21	451.1014 (3.32)	$C_{24}H_{20}O_9$	Unknown	-
**12**	6.20	447.0673 (3.57)	$C_{31}H_{12}O_4$	Unknown	-

Table S5. Characterization of chemical constituents of *Vatairea guianensis* detected on plants leaves by UPLC−ESI−QToF−MS

Peak	*tR*/min	$[M+H]^+$ (mass error, ppm)	Molecular formula	Proposed compound	Ref or database
**1**	5.37	287.0579 (8.36)	$C_{15}H_{10}O_6$	Luteolin	MONA ID 2264<br>GNPS ID 78878
**2**	6.21	271.0613 (2.58)	$C_{15}H_{10}O_5$	Apigenin	MONA ID 2985<br>MONA ID 40003<br>HMDB ID 374058
**3**	6.37	301.0684 (9.30)	$C_{16}H_{12}O_6$	Diosmetin	METLIN ID 44398<br>Campanero et al., 2010[40]<br>Silvestro et al., 2013[41]
**4**	6.97	531.1833 (6.21)	$C_{27}H_{30}O_{11}$	5,3'-dihydroxy-4'-methoxy-7-O-β-glucopyranoside-8-prenyl-isoflavone	Souza et al., 2013[14]
**5**	7.40	355.1180 (0.28)	$C_{20}H_{18}O_6$	Unknow	-
**6**	7.70	385.1296 (2.33)	$C_{21}H_{20}O_7$	Unknown	-
**7**	7.80	369.1360 (5.95)	$C_{21}H_{20}O_6$	3'-methoxy-8-prenylorobol (5,7,4'-trihydroxy-8-prenyl-3'-methoxy-isoflavone)	Souza et al., 2017[16]
**8**	7.99	385.1296 (2.33)	$C_{21}H_{20}O_7$	Unknown	-
**9**	8.22	385.1296 (2.33)	$C_{21}H_{20}O_7$	Unknown	-
**10**	9.00	355.1180 (0.28)	$C_{20}H_{18}O_6$	Luteone	Tahara et al., 1984[42]
**11**	10.03	339.1241 (2.65)	$C_{20}H_{18}O_5$	Lupiwighteone	Souza et al., 2017[16]
**12**	10.43	369.1321 (4.60)	$C_{21}H_{20}O_6$	5,7,3'-triidroxi- 8-prenyl-4'-methoxy-isoflavone	Souza et al., 2017[16]
**13**	11.60	337.1053 (6.82)	$C_{20}H_{16}O_5$	Derrone	Souza et al., 2013[14]

**14**	11.82	367.1157 (6.53)	$C_{21}H_{18}O_6$	5,3'-dihydroxy-4'-methoxy-2",2"-dimethylpyrano-(5",6":8,7)-isoflavone	Piccinelli et al., 2011[43]
**15**	12.23	367.1196 (4.08)	$C_{21}H_{18}O_6$	5,7-dihydroxy-3',4'-methylenedioxy -8-prenyl-isoflavone	Souza et al., 2013[14]

Table S6. Characterization of chemical constituents of *Peperomia pellucida* detected on plants leaves by UPLC−ESI−QToF−MS.

Peak	*tR*/min	$[M+H]^+$ (mass error, ppm)	Molecular formula	Proposed compound	Ref or database
**1**	1.87	565.1553 (0.70)	$C_{26}H_{28}O_{14}$	Isoschaftoside	ReSpect
**2**	2.45	197.1172 (3.04)	$C_{11}H_{16}O_3$	Liolide	MoNA ID 3142433
**3**	2.96	197.0818 (2.02)	$C_{10}H_{12}O_4$	2-Hydroxy-4,6-dimethoxyacetophenone	Soares et al., 2006[26] HMDB ID 29645
**4**	4.06	403.1758 (0.24)	$C_{22}H_{28}O_6$	Unknown	-
**5**	5.61	403.1758 (0.24)	$C_{22}H_{26}O_7$	rel-(7R,8S,70S,80S)-4-Hydroxy-40,50-methylenedioxy-3,5,30-trimethoxy-7,70-epoxylignan	Felippe et al., 2008[27]
**6**	5.69	387.1808 (0.00)	$C_{22}H_{26}O_6$	Pellucidin B	Standard
**7**	5.61	389.1960 (1.02)	$C_{22}H_{28}O_6$	Pellucidin A	Bayma et al., 2000[12]
**8**	5.67	389.1960 (1.02)	$C_{22}H_{28}O_6$	Unknown	-
**9**	8.11	593.2748 (0.50)	$C_{34}H_{40}O_9$	Unknown	-

Table S7. Characterization of chemical constituents of *Phyllantus brasiliensis* detected on plants leaves by UPLC−ESI−QToF−MS

**Peak**	***tR*/min**	**[a][M–H]⁻ or [b][M+H]⁺ (mass error, ppm)**	**Molecular formula**	**Proposed compound**	**Ref or database**
**1**	2.05	289.0706[a] (2.07)	$C_{15}H_{14}O_6$	Catechin[#]	Galaverna et al., 2015[28]
**2**	2.60	289.0706[a] (2.07)	$C_{15}H_{14}O_6$	Epicatechin[#]	Xiao et al., 2017[29]
**3**	3.25	609.1503[a] (7.71)	$C_{27}H_{30}O_{16}$	Rutin[#]	Fu et al., 2016
**4**	3.35	609.1503[a] (7.71)	$C_{27}H_{30}O_{16}$	Unknown[#]	-
**5**	3.92	447.0947[a] (4.47)	$C_{21}H_{20}O_{11}$	Astragalin[#]	He et al., 2013
**6**	4.18	563.1415[a] (2.48)	$C_{26}H_{28}O_{14}$	Unknown[#]	-
**7**	6.39	673.1808[a] (1.03)	$C_{32}H_{34}O_{16}$	*Arabelline	Standard
**8**	5.79	675.1932[b] (1.03)	$C_{32}H_{34}O_{16}$	*4-*O*-*β*-D-apiofuranosyl-(1'''→6")-*β*-D-glucopyranosyldiphyllin	Standard
**9**	6.16	543.1489[b] (2.57)	$C_{27}H_{26}O_{12}$	*5-*O*-*β*-D-glucopyranosyljusticidin B	Standard
**10**	6.25	543.1489[b] (2.57)	$C_{27}H_{26}O_{12}$	*Cleistanthin B	Standard
**11**	6.42	285.0408[b] (3.15)	$C_{15}H_{10}O_6$	Kaempferol[#]	Fu et al., 2016
**12**	6.92	609.1570[b] (–2.29)	$C_{29}H_{30}O_{13}$	*Phyllanthostatin A	Standard
**13**	7.12	513.1400[b] (0.58)	$C_{26}H_{24}O_{11}$	*Tuberculatin	Standard
**14**	9.04	365.1021[b] (1.09)	$C_{21}H_{16}O_6$	*Justicidin B	Standard

*Lignans and [#]Flavonoids

Table S8. Characterization of chemical constituents of *Stryphnodendron pulcherrimum* detected on plants leaves by UPLC−ESI−QToF−MS.

Peak	*tR*/min	$[M+H]^+$ (mass error, ppm)	Molecular formula	Proposed compound	Ref or database
**1**	0.45	377.0880 (1.85)	$C_{18}H_{18}O_9$	Unknown	-
**2**	0.46	169.0148 (6.50)	$C_7H_6O_5$	Gallic acid	METLIN ID 3295<br>GNPS (2014)
**3**	0.46	609.1266 (3.61)	$C_{30}H_{26}O_{14}$	Unknown	-
**4**	0.47	762.1436 (0.52)	$C_{37}H_{31}O_{18}$	Unknown	-
**5**	1.00	609.1266 (3.61)	$C_{30}H_{26}O_{14}$	Gallocatechin-(4β → 8)-gallocatechin	Callemien e Collin, 2007[44]
**6**	1.04	153.0191 (1.96)	$C_7H_6O_4$	Gentile acid	METLIN ID 618
**7**	1.23	137.0241 (1.45)	$C_7H_6O_3$	*p*-Salicylic acid	METLIN ID 3263
**8**	1.23	319.0838 (6.26)	$C_{16}H_{16}O_7$	Unknown	-
**9**	1.23	761.1337 (2.23)	$C_{37}H_{30}O_{18}$	Unknown	-
**10**	1.29	593.1289 (1.01)	$C_{30}H_{26}O_{13}$	Catechin-(4α-8)-gallocatechin	Callemien e Collin, 2007[44]
**11**	1.31	477.1057 (5.03)	$C_{22}H_{22}O_{12}$	Unknown	-
**12**	1.39	305.0663 (0.65)	$C_{15}H_{14}O_7$	Epigallocatechin	GNPS (2014)<br>METLIN ID 44010
**13**	1.41	477.1057 (5.03)	$C_{22}H_{22}O_{12}$	Unknown	-
**14**	1.41	593.1289 (1.01)	$C_{30}H_{26}O_{13}$	Unknown	-
**15**	1.41	761.1393 (5.12)	$C_{37}H_{30}O_{18}$	Unknown	-
**16**	1.78	121.0287 (2.47)	$C_7H_6O_2$	4-Hydroxybenzaldehyde	METLIN ID 62451
**17**	1.78	457.0759 (2.62)	$C_{22}H_{18}O_{11}$	Epigallocatechin-3-O-Gallate	METLIN ID 3550
**18**	1.79	289.0719 (2.42)	$C_{15}H_{14}O_6$	catechin	Galaverna et al., 2015[28]
**19**	1.94	479.0858 (6.67)	$C_{21}H_{20}O_{13}$	Myricetin-3-O-galactoside	HMDB ID 34358
**20**	2.12	197.0466 (8.11)	$C_9H_{10}O_5$	Unknown	-
**21**	2.30	463.0868 (1.94)	$C_{21}H_{20}O_{12}$	Myricitrin	GNPS (2018)

**22**	2.41	447.0923 (0.89)	$C_{21}H_{20}O_{11}$	Quercetin-3-O-deoxyhexoside	GNPS (2018)
**23**	2.44	463.0872 (1.07)	$C_{21}H_{20}O_{12}$	Unknown	-
**24**	2.61	317.0279 (5.67)	$C_{15}H_{10}O_{8}$	Myricetin	METLIN ID 3448<br>HMDB ID 2755
**25**	2.66	301.0344 (1.32)	$C_{15}H_{10}O_{7}$	Quercetin	GNPS (2017)<br>METLIN ID 409<br>HMDB ID 5794
**26**	2.66	431.0984 (1.39)	$C_{21}H_{20}O_{10}$	Unknown	-
**27**	3.06	285.0405 (2.10)	$C_{15}H_{10}O_{6}$	Luteolin	GNPS (2014)<br>METLIN ID 3409

C. Supplementary schemes:

Scheme S1. Isotope standard for peak 1 of *Peperomia pellucida*, calculations of molecular formula and error of mass ppm (Software Masslynx 4.1).

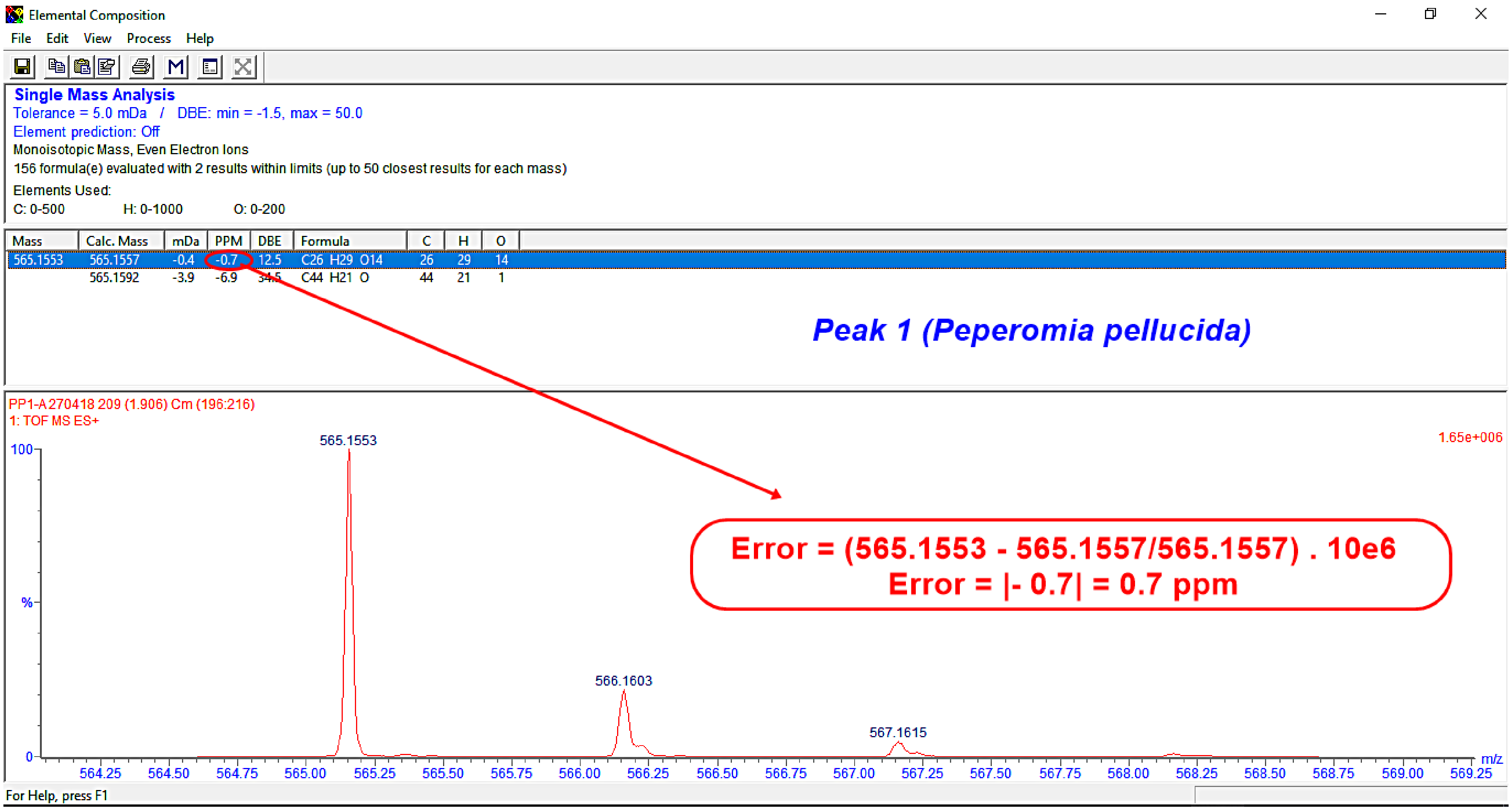

Scheme S2. Isotope standard for peak 3 of *Peperomia pellucida*, calculations of molecular formula and error of mass ppm (Software Masslynx 4.1).

Elemental Composition

File Edit View Process Help

Single Mass Analysis

Tolerance = 5.0 mDa / DBE: min = -1.5, max = 50.0

Element prediction: Off

Monoisotopic Mass, Even Electron Ions

28 formula(e) evaluated with 1 results within limits (up to 50 closest results for each mass)

Elements Used:

C: 0-500 H: 0-1000 O: 0-200

Mass	Calc. Mass	mDa	PPM	DBE	Formula	C	H	O
197.1175	197.1178	-0.3	-1.5	3.5	C11 H17 O3	11	17	3

Peak 2 (Peperomia pellucida)

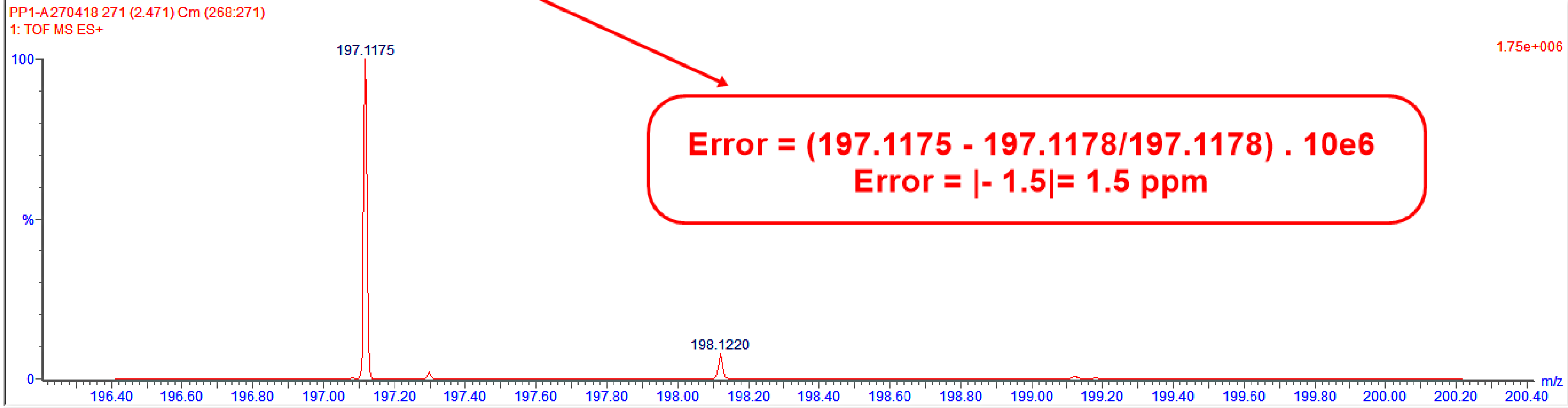

Error = (197.1175 - 197.1178/197.1178) . 10e6
Error = |- 1.5|= 1.5 ppm

For Help, press F1

Scheme S3. Isotope standard for peak 4 of *Peperomia pellucida*, calculations of molecular formula and error of mass ppm (Software Masslynx 4.1).

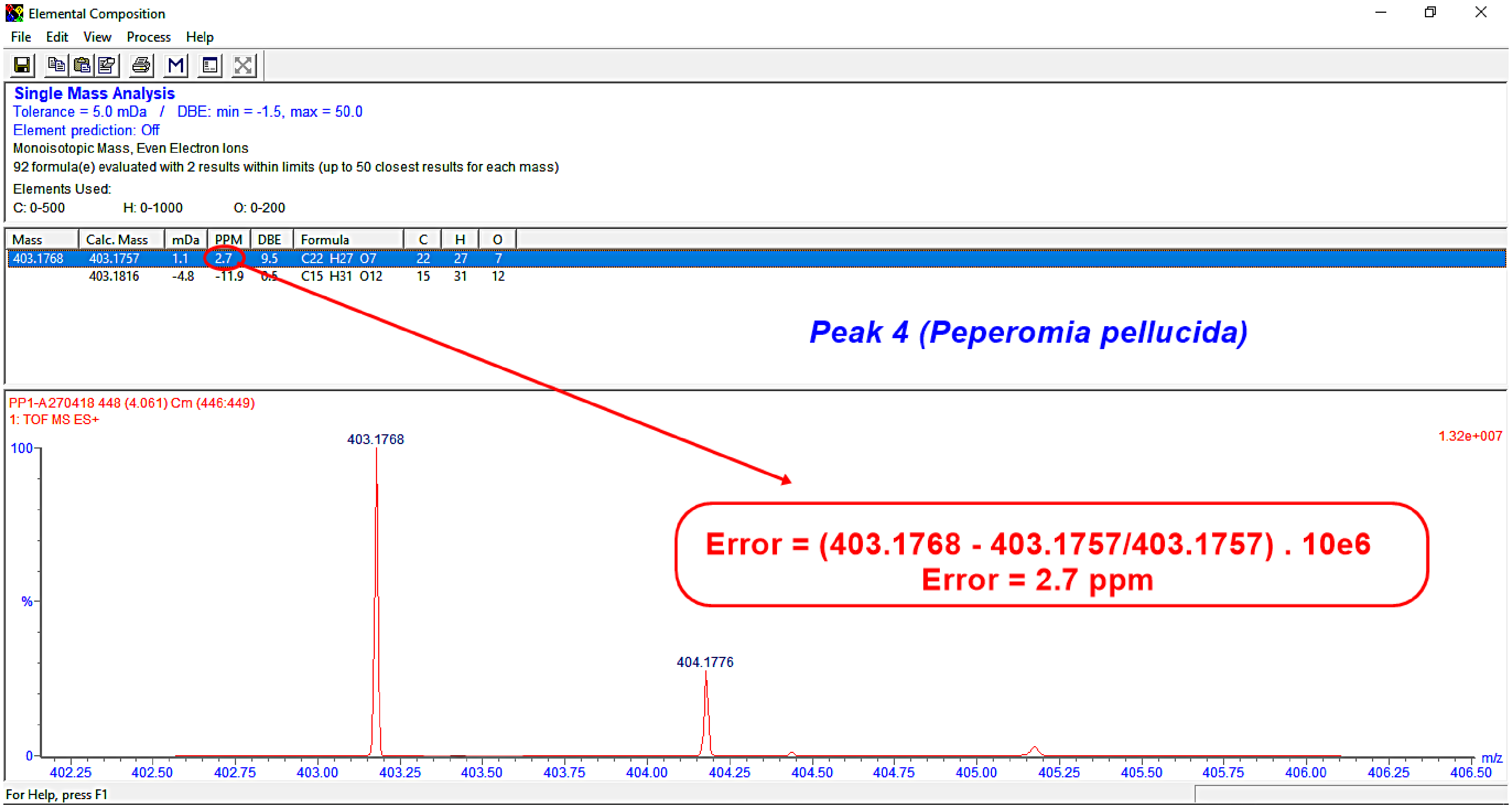

Scheme S4. Example ***(step 1: insert spectrum experimental)*** for peak 2 of *Peperomia pellucida*, calculations of similarity MS/MS in MoNA (https://mona.fiehnlab.ucdavis.edu/).

10/17/2020 MassBank of North America

Quick Search () *Similarity Search ()*

Similarity Search

Please verify your mass spectrum and set your search parameters. Note that this search returns only the 25 highest similarity matches.

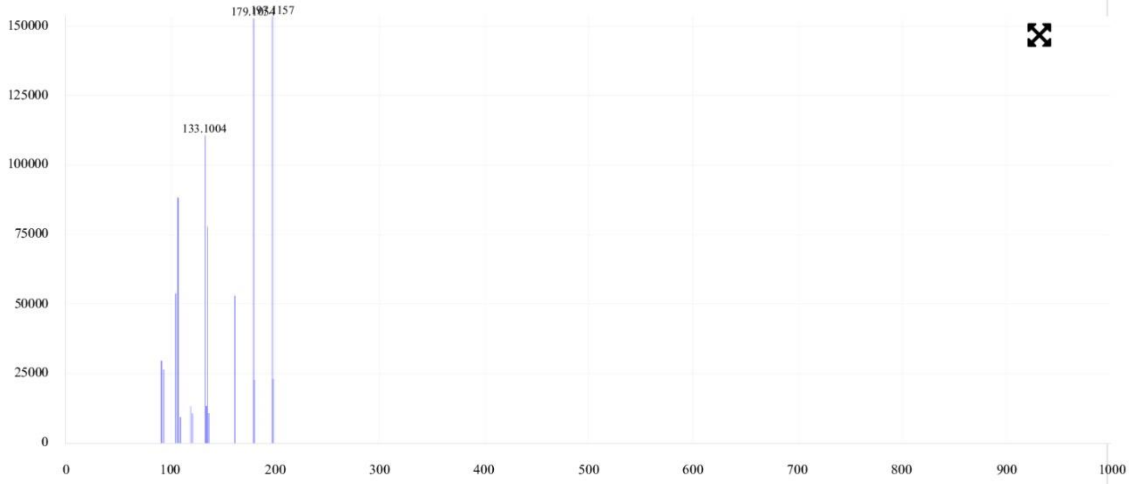

Minimum Similarity (default: 500):

800

Precursor m/z (optional):

197.11

± 5 PPM

Reset | Search for Similar Spectra

Scheme S5. Example ***(step 2: similarity result)*** for peak 2 of *Peperomia pellucida*, calculations of similarity MS/MS in MoNA (https://mona.fiehnlab.ucdavis.edu/).

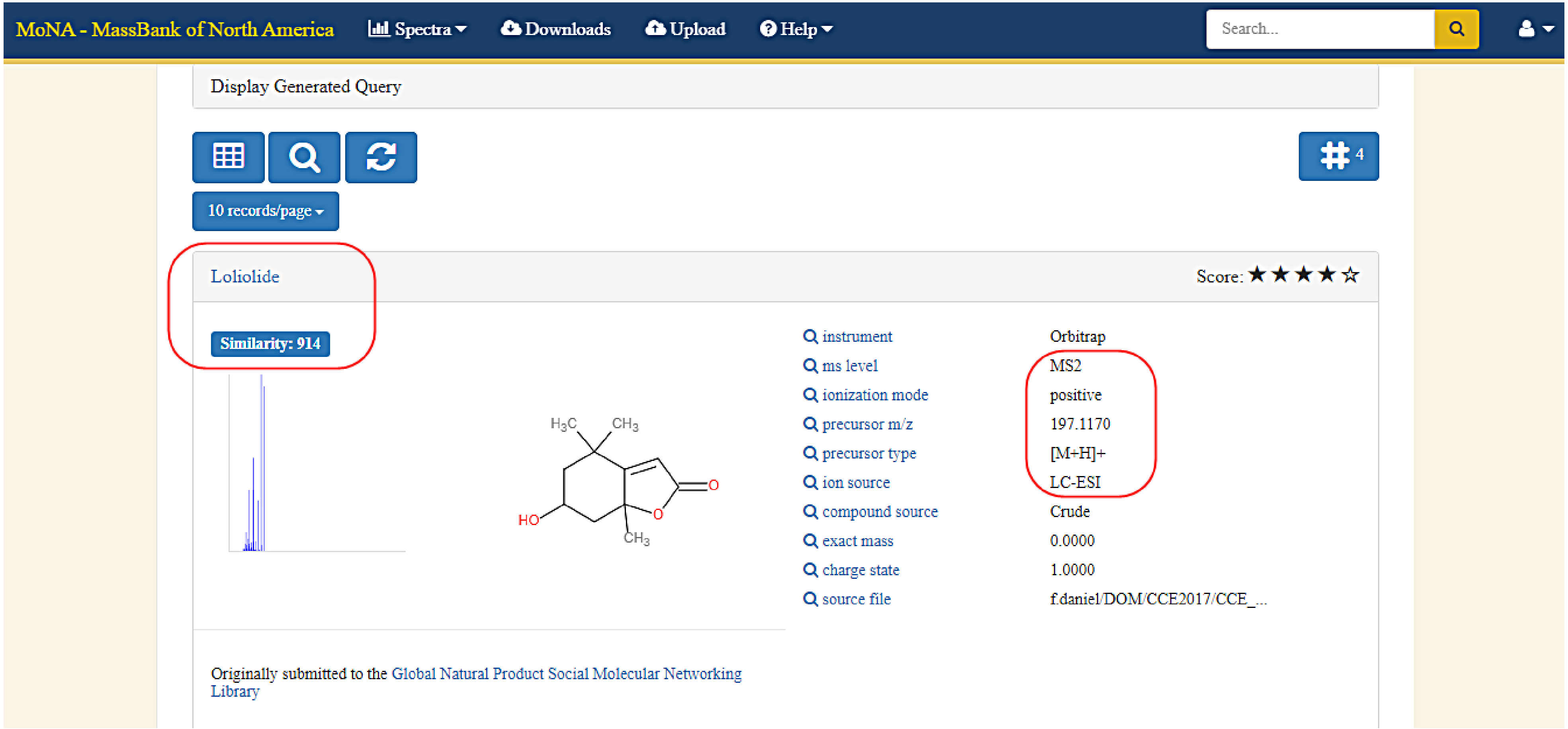

Scheme 6. Example ***(step 3: match of spectrum database and experimental)*** for peak 2 of *Peperomia pellucida*, calculations of similarity MS/MS in MoNA (https://mona.fiehnlab.ucdavis.edu/).

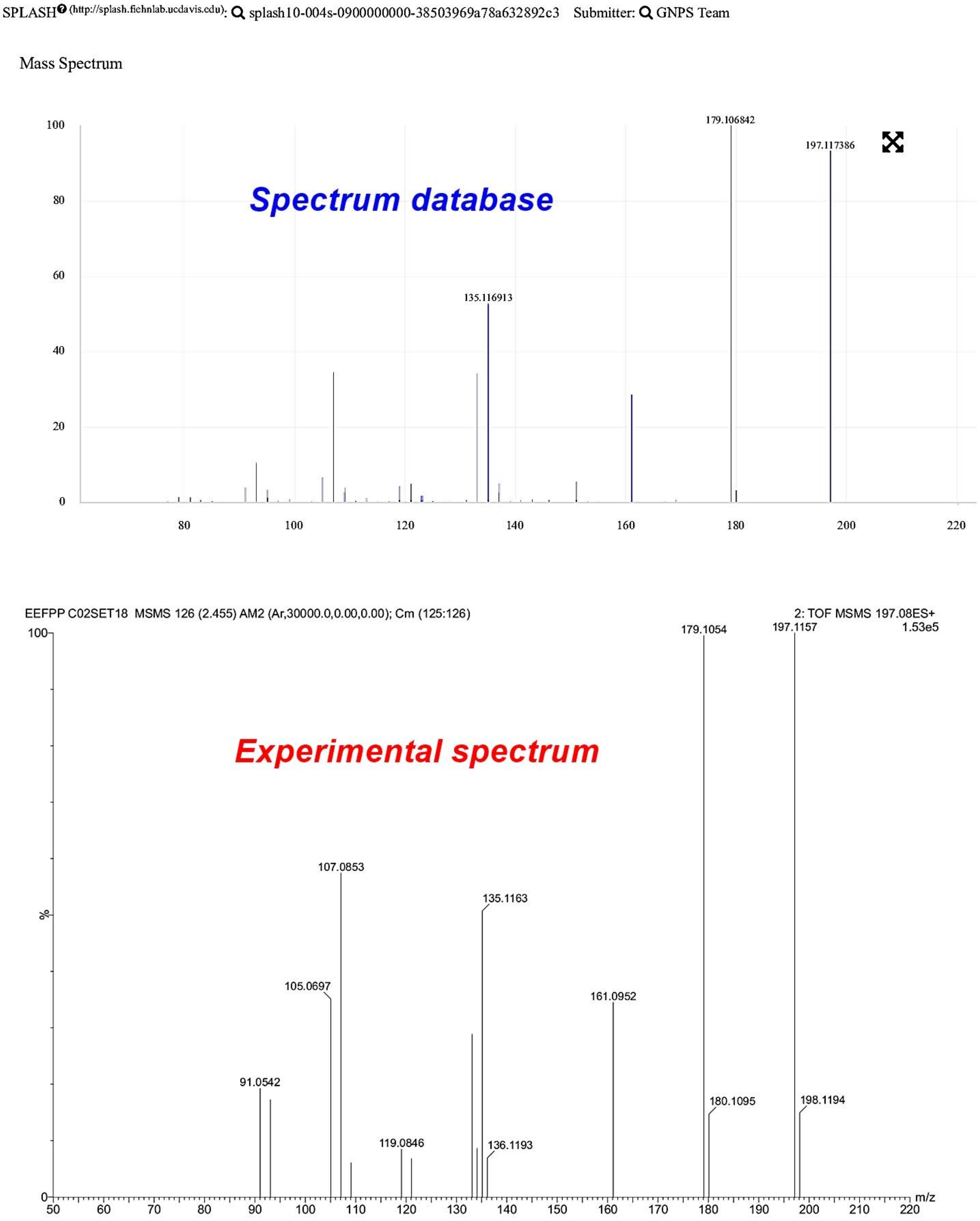

Supplement: Supplementary file 1 [file molecules-25-05992-s001.pdf]
